# Evolutionary, Structural and Functional Interplay of the IκB Family Members

**DOI:** 10.1371/journal.pone.0054178

**Published:** 2013-01-23

**Authors:** Shaherin Basith, Balachandran Manavalan, Vijayakumar Gosu, Sangdun Choi

**Affiliations:** 1 Department of Molecular Science and Technology, Ajou University, Suwon, Korea; 2 Center for In Silico Protein Science, School of Computational Sciences, Korea Institute for Advanced Study, Seoul, Korea; University of South Florida College of Medicine, United States of America

## Abstract

A primary level of control for nuclear factor kappa B (NF-κB) is effected through its interactions with the inhibitor protein, inhibitor of kappa B (IκB). Several lines of evidence confirm the existence of multiple forms of IκB that appear to regulate NF-κB by distinct mechanisms. Therefore, we performed a comprehensive bioinformatics analysis to understand the evolutionary history and intrinsic functional diversity of IκB family members. Phylogenetic relationships were constructed to trace the evolution of the IκB family genes. Our phylogenetic analysis revealed 10 IκB subfamily members that clustered into 5 major clades. Since the ankyrin (ANK) domain appears to be more ancient than the Rel homology domain (RHD), our phylogenetic analysis suggests that some undefined ancestral set of ANK repeats acquired an RHD before any duplication and was later duplicated and then diverged into the different IκB subfamilies. Functional analysis identified several functionally divergent sites in the ANK repeat domains (ARDs) and revealed that this region has undergone strong purifying selection, suggesting its functional importance in IκB genes. Structural analysis showed that the major variations in the number of ANK repeats and high conformational changes in the finger loop ARD region contribute to the differing binding partner specificities, thereby leading to distinct IκB functions. In summary, our study has provided useful information about the phylogeny and structural and functional divergence of the IκB family. Additionally, we identified a number of amino acid sites that contribute to the predicted functional divergence of these proteins.

## Introduction

Nuclear factor kappa B (NF-κB) proteins comprise a family of structurally related and evolutionarily conserved transcription factors that are involved in the control of a large number of normal cellular and organismal processes such as immune and inflammatory responses, developmental processes, cellular growth and apoptosis. The 5 members of the mammalian NF-κB transcription factor family are p65 (RelA), RelB, c-Rel, NF-κB1 (p50 and its precursor p105), and NF-κB2 (p52 and its precursor p100), which associate with each other to form various transcriptionally active homo- and heterodimeric complexes [1], [2]. Studies carried out by Baltimore et al. led to the discovery that NF-κB is regulated through its interaction with inhibitor of kappa B (IκB) proteins [3]. The IκB protein family consists of 11 members: Relish, NF-κB1 (p105), NF-κB2 (p100), Cactus, IκBα, IκBβ, IκBγ, IκBε, IκBζ, IκBNS (also known as IκBδ), and Bcl3. The defining feature of IκB proteins is the presence of multiple (generally 5–8) copies of the ankyrin (ANK) repeats, a 33-residue helix-turn-helix protein motif that is found in many proteins ranging from bacteria to humans [4], [5]. The primary function of IκBs was originally thought to be the suppression of NF-κB activity; however, recent studies have revealed that IκBs are not simple inhibitors of NF-κB activity, but rather pleiotropic NF-κB cofactors and more complex regulators of gene expression [6], [7].

Similar to human p100 and p105, Relish is a *Drosophila* compound protein, comprising an N-terminal Rel homology domain (RHD) and a C-terminal IκB-like region. The NF-κB precursor proteins p100 and p105 contain ANK repeats at their C-terminal region and prior to processing are capable of inhibiting NF-κB activity [8], [9]. In addition, the C-terminal portion of the p105 protein, IκBγ has been reported to inhibit the c-Rel, p50/65 and p50 homodimers [10]. Cactus is a *Drosophila* homolog of mammalian IκB proteins which forms cytoplasmic complexes with NF-κB [11]. In most resting cells, NF-κB dimers associate with one of the typical IκBs such as IκBα, IκBβ and IκBε. These IκBs mask the nuclear localization signal (NLS) of NF-κB, thereby preventing its translocation into the nucleus. The activation of cells with appropriate stimuli, particularly Toll-like receptor ligands or various host immune mediators such as proinflammatory cytokines, including tumor necrosis factor α and interleukin (IL)-1 superfamily proteins [12], [13], [14], [15], lead to the phosphorylation of cytosolic IκBα and its rapid ubiquitin-proteasomal degradation, resulting in the release of NF-κB dimers. These liberated dimers then translocate into the nucleus and bind to κB sites in the promoter/enhancer regions of target genes, resulting in their transcriptional regulation via the recruitment of co-activators and co-repressors. This leads to the expression of primary/early response genes, including 3 atypical/nuclear IκB family members, IκBζ, Bcl3 and IκBNS, which play vital roles in regulating the transcription of secondary response genes by acting as either activators or inhibitors in the nucleus [16]. To date, 11 IκB proteins have been identified.

Generally, individual IκBs associate preferentially with a particular set of NF-κB dimers, thereby modulating the transcriptional response. Cytoplasmic IκBs bind preferentially to NF-κB dimers that possess at least one p65/RelA or c-Rel subunit, thereby masking their NLS and causing them to be retained in the cytoplasm [17]. One of these proteins, IκBβ acts as both a positive and negative regulator [18], and thus has a function similar to that of nuclear IκBs. Among the nuclear proteins, Bcl3 functions as either an activator or an inhibitor of NF-κB in a context-specific manner by regulating its transcriptional activity [19], [20]. IκBNS inhibits the production of IL-6 by associating with NF-κB p50 homodimers, thereby preventing them from binding to the IL-6 promoter [21], [22]. Another member of this group, IκBζ, associates with other nuclear proteins such as STAT3, Brg1 and CEBP1, in addition to NF-κB proteins [2], [23], [24], [25], [26], [27].

A previous study classified IκB proteins into 3 subfamilies and clustered Relish with the IκB proteins, identifying it as an early offshoot of the IκB family [28]. However, because they used only limited sequences and species to construct their phylogeny, it did not provide a full detailed framework for the whole IκB family. The evolutionary history of the IκB family needs to be confirmed by systematic phylogenetic analysis. Furthermore, whether natural selection has driven the evolution of the IκB proteins remains unknown. In this study, we investigated the phylogenetic and molecular evolution of the IκB family more thoroughly. This has allowed us to predict the structural and putative functional motifs and a number of critical amino acid sites that may be significant in the functional divergence of the IκB members. Additionally, principal component analysis (PCA) was employed to characterize the interconformer relationships between experimentally determined ANK repeat domain (ARD) structures. Comparative modeling was also utilized to build 3D structural models of the IκBs, and these structures were subjected to normal mode analysis (NMA) to identify the motion of the domains. These evolutionary, structural and functional analyses of IκB proteins will enable us to understand better the conservation and classification of IκB proteins, as well as providing insights into the evolution of functional divergence between the members of the IκB family.

## Methods

### Sequence collection

In order to identify the IκB homologs, we used 10 biochemically characterized IκB protein sequences from *Homo sapiens* and *Drosophila melanogaster* sources (IκBα_Hs-CAB65556, IκBβ_Hs-AAH15528, IκBε_Hs-NP_004547, Bcl3_Hs-NP_005169, IκBNS_Hs-Q8NI38, IκBζ_Hs-Q9BYH8, NF-κB1_Hs-AAA36361, NF-κB2_Hs-CAC08399, Cactus_Dm-AAA85908, and Relish_Dm-AAB17264) as query sequences. The full protein sequences of the vertebrate and invertebrate species were selected from the NCBI database. Sequences that were not included in NCBI were obtained through PSI-BLAST, ENSEMBL, Uniprot, Interpro and DOE Joint Genome Institute databases. Sequence searches were performed using BLASTP [29] against a nr database [30] with default parameters. Each IκB protein search produced approximately 500 hits. We identified the biochemically characterized proteins manually from the hits and used them as queries for repeated BLAST searches. From the total BLAST output, we selected those IκB homologs with amino acid identity >35% over a stretch of “>X <Y”. The values of “X” and “Y” varied depending upon the specific IκB protein. The lengths of most of the IκBs are quite dissimilar. For instance, IκBα is 317 residues in length, whereas IκBζ is 718 residues long.

Due to these discrepancies in length, we utilized the following specific cut-off values for each IκB protein: IκBα, 250–350; IκBβ, 350–450; Bcl3, 350–550; IκBε, 400–600; IκBNS, 250–400; IκBζ, 600–900; NF-κB1, 850–1100; NF-κB2, 875–1100; Relish, 850–1100; and Cactus, 400–600 residues. We eliminated all hypothetical, putative, predicted, redundant and unnamed proteins from our dataset. This cut-off analysis identified 545 sequences from a list of sequences for 172 unique species; the sequences were subjected to SMART, Pfam [31] and InterPro [32] analyses to identify the domain architecture. On the basis of this analysis, we refined the search results manually to further reduce hits with partially conserved functional domains and other false positives and finally selected the remaining 386 sequences from 124 unique species for further analysis. Pairwise sequence comparisons of all of the IκB proteins were carried out using the Needleman-Wunsch alignment program from the EMBOSS package [33], [34].

### Sequence alignments and phylogenetic analysis

MSA is the second critical step in phylogenetic analysis and the alignment quality may have an enormous impact on the final phylogenetic tree. All IκB sequences (vertebrate, invertebrate and whole IκBs) were imported into the Geneious Pro software v5.5.7 (Available from http://www.geneious.com/), where the initial alignment was performed using the plugin MAFFT v6.814b [35] used in Geneious software with default parameters (auto algorithm; scoring matrix = BLOSUM62; gap open penalty = 1.53; and offset value = 0.123). The MSAs were manually inspected using Jalview [36]. A few particularly gap-rich positions, poorly aligned and divergent regions from the alignments were excluded from the phylogenetic analysis. The final dataset contained 340 sequences from 111 organisms (Table S1). The MSAs were used to construct 3 phylogenetic trees by using 2 different and independent approaches: the NJ and Bayesian methods. The NJ and Bayesian phylogenetic tree reconstructions were performed using Geneious Pro v5.5.7. The molecular distances between the aligned sequences were calculated using the Jukes-Cantor genetic distance model. All gaps and missing data in the alignments were accounted for by pairwise deletions. Branch points were tested for significance by bootstrapping with 1000 replications. Bayesian analysis was conducted using the MrBayes v3.1.2 [37] plug-in implemented in Geneious. The MrBayes parameters were as follows: prset aamodelpr = mixed; lset rates = gamma; Ngammacat = 4; mcmcngen = 1100000; samplefre = 200; nchains = 4; and starting tree = random. Branch points were tested for significance by bootstrapping with 1000 replicates.

### Homology modeling

The modeling procedures used in the current study have been described previously [2], [38], [39], [40]. A homology model of IκBε was built using the top 2 ranked templates (Bcl3 (1K1A) and IκBβ (1K3Z)), whose sequence identities were 42% and 43.89%, respectively. In the case of IκBNS and IκBζ, models were built using Bcl3 (1K1A), which shared the highest sequence identity with the 2 target sequences (36.4% with IκBNS and 37.28% with IκBζ), as a single template. IκBα (1IKN) served as a suitable template for Cactus. The sequence identity of Cactus with IκBα was 33%. Another ARD, 1N11, served as a suitable template for Relish, whose sequence identity was 20%. Bcl3 (1K1A) acted as a template for both NF-κB1 and NF-κB2, whose sequence identities were 40% and 39%, respectively. The target-template sequence alignments were performed using MUSCLE [41]. All of the models were built using Modeller 9v8 [42]. We constructed 3D models using a distance restraint algorithm based on the MSA of the target sequence with the template structures by applying the CHARMM force field [43]. An optimization method, which involved conjugate gradients and MD-simulated annealing, was employed to minimize violations of spatial restraints. For model building, the default parameters included in the “automodel” class were used. Subsequently the 20 models were subjected to automatic loop refinement, from which the best final model was selected based on stereochemical and energetic evaluations. The model was further evaluated with DOPE (Discrete Optimized Protein Energy), a pairwise atomic distance statistical potential that assesses atomic distances in a model relative to those observed in many known protein structures [44]. DOPE is based on an improved reference state which corresponds to the non-interacting atoms in a homogeneous sphere with the radius dependent on a sample native structure. It accounts for the finite and spherical shape of the native structures. The stereochemical quality of these proteins was assessed using ProQ [45] and MetaMQAP [46]. The final models were subjected to energy minimization using the AMBER 03 force field implemented in YASARA [47].

### Functional divergence

Type I and II functional divergence among the IκB subfamilies was examined using the DIVERGE 2.0 software [48], [49], which implements the tests suggested by Gu [37] that can be used to determine whether the coefficients of divergence (*θ_I_* and *θ_II_*) are significantly greater than 0. The IκB MSA was used as an input in DIVERGE 2.0, and a rooted NJ tree was generated using the Poisson correction distance measure. This tool utilizes the phylogenetic tree to assess site-specific changes in evolutionary rates within amino acid alignments when comparing subclades. Using type I functional divergence, the coefficient of evolutionary functional divergence (*θ*) can be measured to assess the changes in site-specific evolutionary rates, i.e., *θ* = 0 indicates no functional divergence, while increasing values indicate increasing functional divergence, with *θ* = 1 being the maximum. Utilizing *θ*, we tested for significant functional divergence (LRT, *p*<0.05) for each of the pairwise comparisons of the 10 different IκB subfamilies, which were subdivided into taxonomic groups (1 or 2 representative sequence from each species class), where appropriate, based on the phylogenetic tree. For instance, the IκBα sequences were divided into mammalian IκBα (*Homo sapiens and Mus musculus*), avian IκBα (*Gallus gallus*), reptilian IκBα (*Anolis carolinensis*), amphibian IκBα (*Xenopus tropicalis*), and actinopterygian IκBα (*Latimeria chalumnae*) sequences. Using type II functional divergence, residues with radical biochemical changes between the subfamily groups can be identified. We focused on radical changes among the vertebrate IκB subfamilies and applied a cut-off value of *θ*>17 for site-specific posterior probabilities. Type II functional divergence was analyzed for the following 8 IκB subfamilies: IκBα, IκBβ, IκBε, IκBNS, IκBζ, Bcl3, NF-κB1 and NF-κB2.

### Analysis of selective pressure

The detection of selective forces acting at a single amino acid site in a given protein sequence may be of importance in understanding the processes underlying the evolution of that particular protein. We carried out site-wise Ka/Ks analysis of IκB gene coding sequences using the Selecton server version 2.4 [50], [51], an online program to detect the selection forces acting at each amino acid site in the IκB sequences. The Selecton server uses a non-aligned file containing homologous coding DNA sequences or a codon-aligned file of coding DNA sequences in FASTA format as the input. We submitted the aligned IκB gene coding sequences individually for each IκB subfamily member to Selecton as the input. Among these coding sequences, IκB representative sequences were used as the query. Subsequently, we used the Selecton server to analyze the entire IκB coding sequences and identify the residues involved in strong purifying selection.

### PCA of ARD crystal structures

Using the 3D atomic coordinates of Bcl3 as the query, we identified homologous structures in the PDB by using minimum cut-off values of 20% sequence identity and 40% coverage. In this analysis, 71 solved structures were identified. PCA was employed to examine interconformer relationships; this method has been described in detail elsewhere [52]. This analysis allowed us to view the distribution of the dataset structures in the subspace spanned by PC1 and PC2, and therefore to discriminate or cluster the conformations based on their most distinctive structural similarities or dissimilarities. The analysis was performed with software from the ProDy package [52], [53].

### Normal mode calculations

The anisotropic network model (ANM) is a coarse-grained model for the protein dynamics, which is used to investigate the vibrational motions [54], [55]. It uses elastic network methodology (ENM) and represents the system in residue level. Each node is represented by Cα atom of a residue and the overall potential is the sum of the harmonic potential between the interacting nodes. Each pair of residues separated less than a cutoff distance is assumed to be connected by harmonic spring. Information about the orientation of each interaction with respect to the global coordinate system is considered within the Force constant matrix and allows prediction of anisotropic motions.

A Hessian matrix can describe the force constant of the system. If the native structure corresponds to the energy minimum, then we can expand the potential energy in the Taylor series in terms of small positional deviations of residues ΔR from the mean equilibrium values R_Ο_

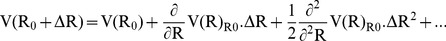
(1)


For small deviations one can neglect terms higher than the second. Taking V(R_Ο_) = 0, and noticing that the first derivative is equal to zero, we can rewrite the above equation as:
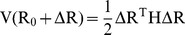
(2)


Where H is the matrix of second derivatives. If the distance between two residues i and j is less than or equal to a cutoff value (in this study, we maintained the cut-off distance (r_c_) between the Cα atoms as 15 Å), then we can calculate H_3i+k, 3j+l_ (K, l = 1–3) by applying harmonic potential in the computation of the second derivative; otherwise it is set to 0.

The Hamiltonian of the system can be written as:
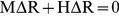
(3)


From the assumption of the harmonic nature of oscillations, we can express the Hessian as:
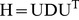
(4)


Where U is a matrix of eigenvectors, and D is a diagonal matrix of eigenvalues.

3N-6 non-zero eigenvalues can be calculated according to:
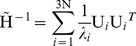
(5)


Where λ_i_ are the eigenvalues of H sorted by their size from small to large and U_i_ the corresponding eigenvectors. The eigenvectors describe the vibrational directions and relative amplitude in the different modes.

The mean square fluctuation of individual residues can be obtained by summing the fluctuations in the individual modes as follows:
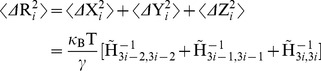
(6)


ProDy and the ANM were used to obtain simulated functional motions from the IκB structures [52], [53], [55].

## Results

### Domain organization and superimposition of IκBs

The domain organization of each IκB protein family member is shown in Figure S1A. All IκB proteins possess common domain architecture, such as an N-terminal region followed by a C-terminal ARD. Proteins in the NF-κB superfamily of transcription factors are related through a highly conserved N-terminal RHD, which contains sequences required for DNA binding, dimerization, and nuclear localization [56], [57]. In most cases, NF-κB proteins have C-terminal IκB-like inhibitory domains consisting of ANK repeats, which must be removed for their activation.

The number of ARDs varies among these IκBs, which may be important for determining their binding specificity. In cytoplasmic IκBs (IκBα, IκBβ and IκBε), the N-terminal regulatory region contains phosphorylation and ubiquitination sites for signal-dependent degradation and nuclear export signals that contribute to their observed cytoplasmic localization. Subsequently, the C-terminal ARD plays a pivotal role in their physical interaction with Rel/NF-κB subunits, and thereby modulates the cellular responses [2], [17]. The C-terminal region to the ARD in cytoplasmic IκBs is rich in proline, glutamic acid, serine, and threonine (PEST) residues, forming a so-called PEST motif, which is indispensable for interactions with the NF-κB dimer and its subsequent removal from DNA [58]. The PEST motif is common among proteins that display a rapid turnover in cells [59]. To check whether all IκB family members contain the PEST motif, we utilized ePESTfind from the EMBOSS package [33], [34]. We found that the cytoplasmic IκBα, IκBβ, Relish, Cactus and Bcl3 proteins contain a PEST motif at their C-terminal end, whereas IκBζ and IκBNS possess a PEST motif in their N-terminal region, and the remaining IκBs do not possess any PEST motif. Unlike cytoplasmic IκB PEST motifs, the PEST motif of nuclear Bcl3 undergoes phosphorylation of its serine residues during post-translational modification, causing it to have a function similar to that of cytoplasmic IκBs [2], [60], [61], [62]. No functional studies of the N-terminal PEST motif are currently available; hence, future biochemical studies are required to clarify its function.

Generally, the ANK repeat is a 33-amino acid consensus sequence motif in our constructed models. This motif forms 2 anti-parallel α-helices, followed by a loop of variable length at a right angle. Each repeat begins and ends with short hairpin turns that protrude away from the α-helix. This non-globular fold is stabilized by intra- and inter-repeat hydrophobic interactions. In Figures S1B and C, we show the structural superimposition of typical IκB proteins (IκBα, IκBβ, IκBε, IκBζ, IκBNS and Bcl3) and IκB-like domain containing proteins (Cactus, Relish, NF-κB1 and NF-κB2) separately. The structural superimposition of IκBs (Figures S1B and C) show that the regions between the first, fourth and fifth ANK repeats are oriented differently and do not align with those in the rest of the IκB members. The major deviation among the IκB members mainly occurs in the finger loop region of ANK1, ANK3, ANK4, ANK5 and ANK6 (Figures S1B and C, marked with a star). Additionally, minor deviations were observed within and between the ANK repeats. This shows that structural variation in the finger loop region might be responsible for the binding specificity of IκBs. This hypothesis is supported by our PCA analysis of the ARD, as described below, which showed that all ARD-containing proteins were structurally similar and major conformational changes took place only in the finger loop region, indicating that it is primarily responsible for their functional divergence.

### Identification and sequence analysis of IκB family members

Biochemically characterized IκB representative sequences were retrieved from the NCBI database. We used these sequences as queries to search other non-redundant (nr) databases (detailed in the Materials and Methods section) and identified 386 homologous proteins in vertebrates and invertebrates. All-against-all pairwise sequence alignments were carried out for all 386 sequences to determine the inter- and intra-similarity across the whole IκB family. Inter-subfamily similarity values varied from 4% to 82% and intra-subfamily similarity values ranged from 12% to 99.7%. The sequence identity within the IκB family members ranged from 4% to 99.7% (Table 1), suggesting that the gene duplications that gave rise to them are relatively ancient. Due to these ancient genome duplication events, we observed several IκB homologs in different species.

**Table 1:** Inter- and intra-group sequence similarities among the IκB family members.

IκB proteins	IκBα	IκBβ	IκBε	IκBNS	IκBζ	Bcl3	Cactus	Relish	NF-κB1	NF-κB2
IκBα (67)	32.8–99.7	11.2–31.2	15.2–33.5	12.4–24.6	7.8–21.4	6.8–21.8	16.9–29.4	6.7–18.6	7.0–20.9	5.1–14.3
IκBβ (36)	14–29.1	25.4–98.6	14.6–29.3	13.7–22.6	4.9–19.2	7.9–27.6	15.3–24.8	6.8–15.3	5.8–20.0	4.7–15.6
IκBε (37)	8.2–24.2	8.9–24.5	24.9–97.4	15.2–25.4	11.7–22.7	6.4–25.1	13.8–22.0	5.8–18.9	8.8–23.4	7.6–17.1
IκBNS (39)	10.8–23.2	9.1–23.4	13.1–29.0	34.6–85.1	13.5–38.5	8.7–25.2	12.9–20.5	6.1–16.0	6.6–18.6	8.1–13.4
IκBζ (41)	5.3–13.5	8.2–15.1	9–21.2	11.5–22.6	20.9–99.3	6.2–15.1	9.0–20.2	6.4–17.7	6.9–19.1	6.2–19.9
Bcl3 (32)	8.5–22.3	9.8–26.3	18–29.6	12.2–25	5.8–18.1	12–98.7	14.6–22	8.9–17.7	7.8–22.4	5.9–16.3
Cactus (19)	8.2–24.7	8.9–24.8	13.8–25.1	12.2–19.3	10.8–21.7	5.8–22	14.5–94.2	9.6–18.8	7.2–20	6.2–15.5
Relish (36)	3.6–9.6	6.2–10.7	5.8–13.1	5.7–11.0	4.4–18.5	4.7–11.1	6.5–11.6	13.7–75	7.1–29.9	6.0–24.2
NF-κB1 (42)	4.2–12.9	5.1–12.6	7.8–14.7	7.2–14.4	5–18.1	7–13.8	6.9–13.8	12.9–31.6	15.8–98.8	10.2–82
NF-κB2 (37)	5.9–12.8	5.9–13.4	7.9–14.9	8.6–15.4	6.8–18.7	7–16.2	7.2–14.3	11.3–29.3	8.7–42.6	25.3–98.7

All-against-all pairwise sequence comparisons were carried out using the Needleman-Wunsch algorithm implemented in the EMBOSS package. The numbers in parentheses next to the names of the IκB family members indicate the number of sequences included in our analysis for each subfamily.

**Figure 1:**
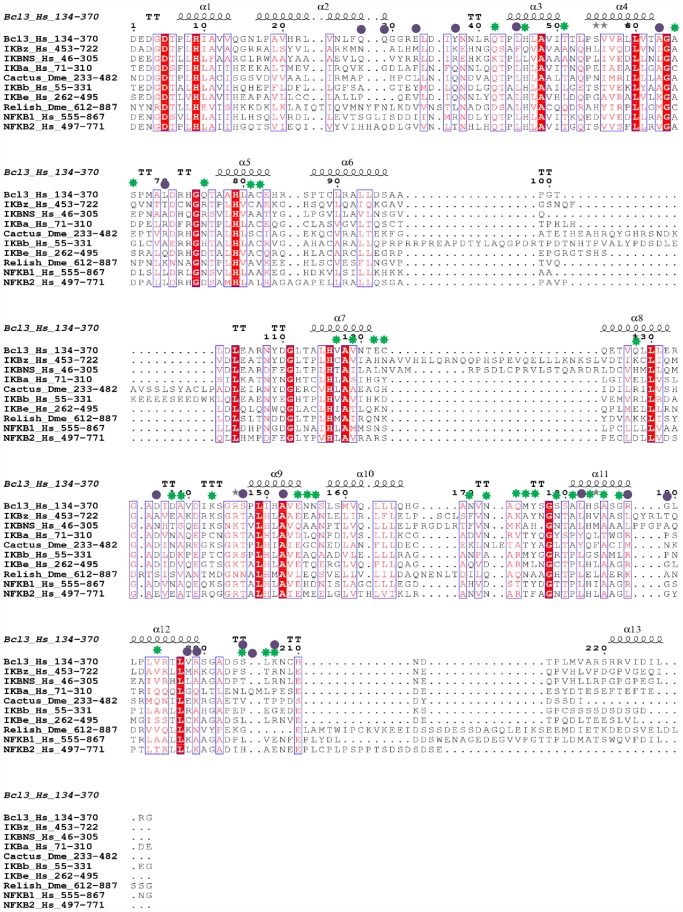
Sequence comparison of IκB ARDs. MSA of the ARDs from the representative IκB family members along with the Bcl3 ARD crystal structure. The amino acid numbers corresponding to the ARD regions for each representative sequence are shown beside each IκB protein name. The residues involved in type I and II divergence are marked in green 7-point stars and purple circles, respectively. The highly conserved regions in the sequence alignment of IκB ARDs are represented in blocks. At the top of the sequence alignment, the secondary structure prediction in relation to the structure of Bcl3 is shown.

In order to identify the similarities or variations among the IκB ARDs, we took representative sequences from each clade and performed multiple sequence alignment (MSA) by using the MAFFT (rapid MSA tool based on Fast Fourier Transform) software [35]. We observed that large gaps or improper alignments were mainly found in the N-terminal region (data not shown); therefore, we trimmed off the N-terminal region and used only the C-terminal ARD for further MSA (Figure 1). In this analysis, we observed the following large insertions between or within the ANK repeats: (i) IκBβ contains a 41-residue insertion between ANK3 and 4; (ii) Cactus contains a 31-residue insertion between ANK3 and 4; (iii) IκBζ contains a 27-residue insertion within ANK4; (iv) Relish contains a 13-residue insertion within ANK 1; and (v) IκBNS contains a 20-residue insertion within ANK4. In addition to the MSA of IκB representative sequences, an alignment of all IκB homologs was conducted, which clearly showed that the functional regions of each IκB protein were highly conserved across species, indicating similar roles in different species (data not shown).

### Phylogenetic analysis: 3 robust IκB protein subfamilies

The available IκB sequences were retrieved from the major sequence databases. Querying major databases with the full-length representative sequences from the 10 IκB subfamilies identified 545 homologous proteins in vertebrates and invertebrates. On the basis of the filtering criteria, a few sequences were discarded and the final dataset included 340 sequences from 111 species that were subjected to phylogenetic tree reconstruction (Table S1). The final dataset (340 sequences) included 64 IκBα, 35 IκBβ, 36 IκBε, 25 Bcl3, 32 IκBNS, 38 IκBζ, 14 Cactus, 25 Relish, 36 NF-κB1 and 34 NF-κB2 sequences. To explore the phylogenetic relationships among the IκBs, we constructed a rooted tree by utilizing the neighbor joining (NJ) and Bayesian methods for the final dataset derived from 111 species. The results obtained from these 2 phylogenetic methods produced similar tree topologies.

**Figure 2:**
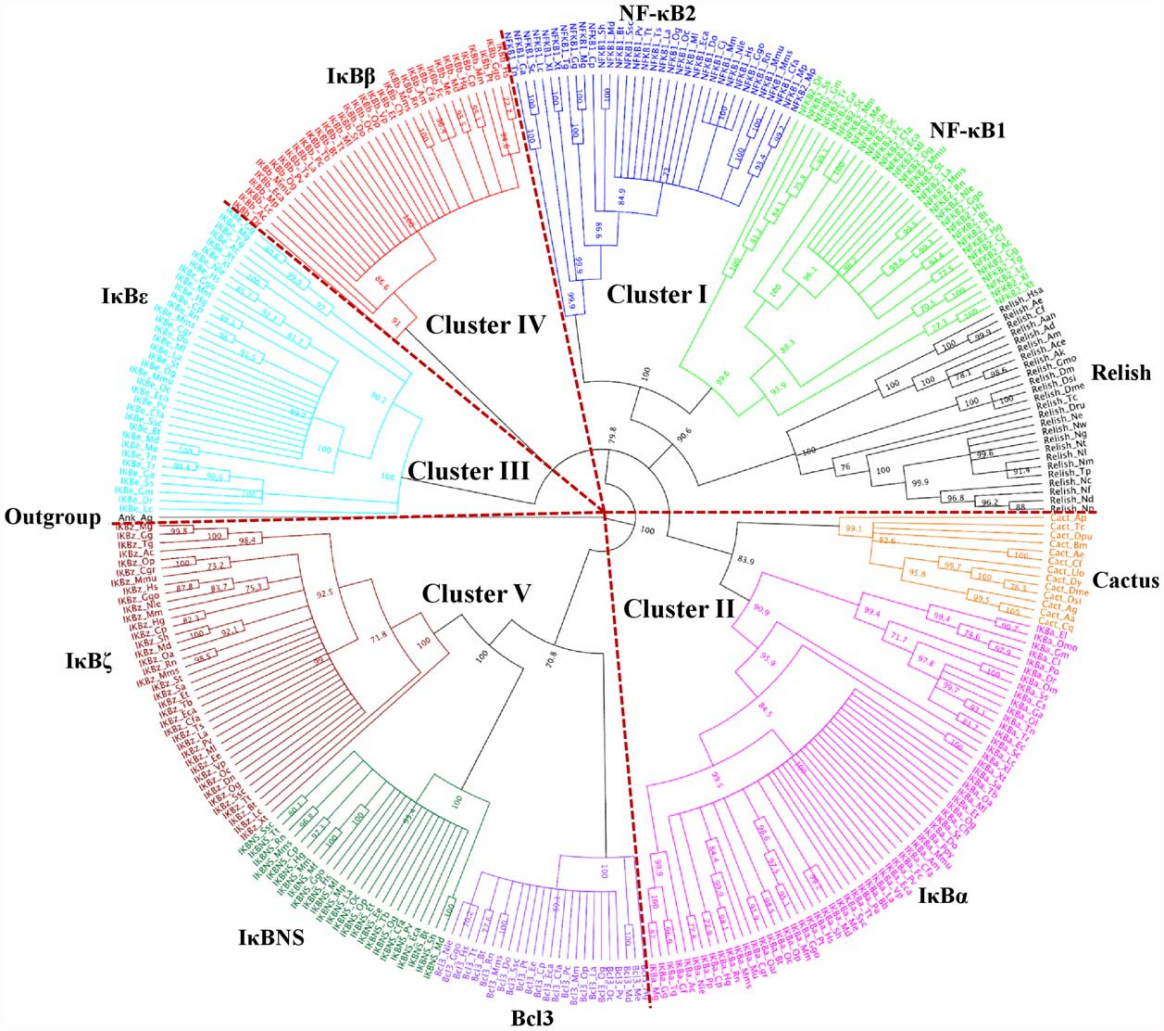
Phylogenetic relationships among all IκB family members determined using the NJ method. A total of 328 protein sequences were included in this analysis. Bootstrap scores higher than 70% have been provided. The sponge *Amphimedon queenslandica* was considered as an outgroup. The clustering of IκB family members into 5 major clades is shown. Each IκB member is represented by a unique color in the phylogenetic tree: IκBα (magenta), IκBβ (red), IκBε (cyan), IκBNS (dark green), IκBζ (brown), Bcl3 (purple), Cactus (orange), Relish (black), NF-κB1 (blue) and NF-κB2 (light green). Taxa terminologies are presented as the IκB protein name followed by an abbreviated form of the species name. Please refer to the Results section and Table S1 for their description and species names, respectively.

In the phylogenetic tree reconstruction for all of the IκB sequences (328 sequences), the sponge *Amphimedon queenslandica* (GenBank ID: XM_003387518.1) was considered as an outgroup. This species has 2 protein sequences with ANK repeats that appear to be IκB proteins; the C-terminal repeats of its NF-κB protein are approximately 40% identical to those of human NF-κB proteins, and the ANK repeats of another gene are also quite similar (40–45% identity) to human IκBα and Bcl3 as well as to *Nematostella vectensis* IκB and Bcl3 [57]. The localization of the last internal branch of the NJ-distance tree with bootstrap values above 70% allowed 5 major clades or clusters among the IκB proteins to be distinguished in the first approach in addition to the *A. queenslandica* outgroup, each of which is shown in a unique color (Figure 2). These major clades included (i) Relish with NF-κB1 and NF-κB2 (90.6% bootstrap value); (ii) Cactus with IκBα (83.9% bootstrap value); (iii) IκBε (100% bootstrap value); (iv) IκBβ (91% bootstrap value); and (v) Bcl3 with IκBζ and IκBNS (nuclear IκB proteins; 70.8% bootstrap value).

It is interesting to study the topologies inside these major clades. According to the distance tree, clade I consisted of 2 groups/subfamilies, i.e., the Relish group and the NF-κB1 and NF-κB2 group. Our distance analysis clearly clustered Relish with the other IκB proteins and identified it as an early offshoot, thereby suggesting that Relish is the ancestor of the IκB family. Moreover, since Relish clustered with the NF-κB paralogs, it can represent a direct arthropod homolog of the NF-κB genes. The second group represents typical paralogous genes, NF-κB1 and NF-κB2 that descended from the duplication of a unique ancestral gene. In clade II of the distance tree, the Cactus gene represents the homolog of the IκBα gene. Cactus also represents a direct arthropod homolog of the IκBα genes in a similar manner to Relish. The branching order of the IκBα orthologs was well supported by the bootstrap values and fits well with the evolution of the species (Osteichthyes < Amphibia < Reptilia < Aves < Mammalia). However, this method separated IκBε and IκBβ into 2 distinct clades (clades III and IV). Moreover, it remains unclear from the phylogenetic tree whether these 2 subfamilies are more or less closely related to any other member of the IκB family. Clade V constitutes the nuclear IκB proteins that were clustered into 2 groups, among which Bcl3 forms the first group and IκBζ and IκBNS form the second group. From clade V, it is apparent that the IκBNS gene represents the direct homolog of the IκBζ gene. Moreover, it is clear that vertebrate-specific gene duplication gave rise to IκBNS and IκBζ, which are linked together with a 100% bootstrap value. The overall topology of the whole IκB phylogeny tree was well supported by high bootstrap values ranging from 70% to 100%. Additionally, the branching order of the IκB subfamily members fits well with the evolution of the species.

**Figure 3:**
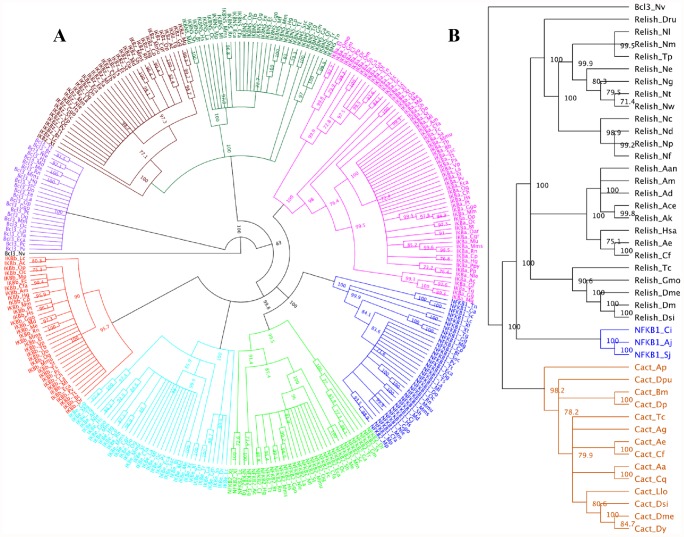
Phylogenetic relationships among vertebrate and invertebrate IκB members determined using the NJ method. (A) A total of 297 vertebrate protein sequences were included in this analysis (IκBα, IκBβ, IκBε, IκBζ, IκBNS, Bcl3, NF-κB1 and NF-κB2). (B) A total of 43 invertebrate protein sequences were included in this analysis (Cactus, Relish and NF-κB1). Bcl3 from *Nematostella vectensis* was considered as an outgroup. Bootstrap scores of >70% have been provided. Each IκB member is represented by a unique color in the phylogenetic tree: IκBα (magenta), IκBβ (red), IκBε (cyan), IκBNS (dark green), IκBζ (brown), Bcl3 (purple), Cactus (orange), Relish (black), NF-κB1 (blue), and NF-κB2 (light green). Taxa terminologies are presented as the IκB protein name followed by an abbreviated form of the species name. Clade organizations obtained for both vertebrate and invertebrate phylogenies are identical to all IκB phylogeny.

In order to validate the clustering organization of the IκB family members (Figure 2), we constructed IκB vertebrate (297 sequences) and invertebrate (43 sequences) phylogenetic trees (Figures 3A and B). Interestingly, these 2 trees produced a clade organization identical to that of the whole IκB phylogeny tree. In both IκB vertebrate and invertebrate tree reconstructions, *N. vectensis* Bcl3 was considered as an outgroup. Additionally, all of the subclades in the vertebrate and invertebrate phylogenetic trees had significant bootstrap values ranging from 70% to 100%.

The evolution of the IκB genes in each of these clades recapitulates the phylogeny of the species. The Relish and Cactus genes were only present in invertebrates (Arthropod and Mollusca lineages), whereas the NF-κB1 and Bcl3 genes were present in vertebrate and invertebrate lineages. Remarkably, *N. vectensis*, the lowest invertebrate species, appears to have a genuine Bcl3 gene. The sporadic appearance of Bcl3 throughout evolution may be due to its distinct properties among the IκB protein family [57]. Except for IκBε, the other IκB genes, including NF-κB2, IκBα, IκBβ, IκBNS and IκBζ are only present in vertebrate lineages. During sequence searching, we identified one invertebrate nematode species (*Trichinella spiralis*; NCBI accession ID XP_003377889) in the IκBε subfamily. However, we did not include this sequence in our analysis due to our length filtering criteria. Most of the vertebrates examined have exactly one gene orthologous to each of the IκBs. However, there are a few exceptions in Aves, which lack the IκBβ and Bcl3 genes; Reptilia, which lack the NF-κB1 gene; and Amphibia, which lack the IκBβ gene. Taken together, these observations clearly indicate that the 8 mammalian IκB proteins arose due to the requirements for IκB proteins with distinct affinities for different NF-κB transcriptional regulatory processes.

From our data, it is clear that most of the mammalian IκB genes have been duplicated, and that the copies diverged from each other prior to the divergence of the earliest mammalian lineage. Some of the non-mammalian vertebrates appear to have phylogenetic affinity with some of the mammalian lineages, with significant bootstrap support. This is particularly evident for several proteins from Aves, Amphibia, Reptilia, and Osteichthyes (fishes) that tend to branch with their mammalian orthologs. Taken together, these observations clearly indicate that many of the IκB-like genes must have duplicated prior to the Mammalia-Aves, Mammalia-Amphibia, Mammalia-Reptilia, and Mammalia-Osteichthyes divergences. Finally, non-mammalian genomes contain noticeably fewer IκB subfamily members when compared with mammalian genomes, indicating that the mammalian genome utilizes multiple NF-κB transcriptional regulatory processes.

In conclusion, the IκB family can be divided into 3 robust subfamilies according to their structural, domain and clade organization: (i) Relish, NF-κB1 and NF-κB2 proteins, which are characterized by the presence of an RHD in their N-terminal regions and ANK repeats in their C-terminal regions; (ii) Cactus, IκBα, IκBβ and IκBε proteins, which are characterized by the presence of 6–7 ANK repeats; and (iii) the inducible nuclear IκB proteins IκBζ, IκBNS and Bcl3, which are characterized by the presence of 7 ANK repeats (Figure S1A). Our current phylogenetic analysis using different methodologies suggests that the IκB subfamilies might have diverged and been duplicated from a unique ancestral set of ANK repeats that had acquired an RHD, i.e., Relish before any duplication. Further, this analysis identified Relish as the earliest lineage and the presence of only few paralogous genes (NF-κB1 and NF-κB2; IκBζ and IκBNS) within the IκB subfamilies.

### Sites contributing to type I and II functional divergence among the IκB subfamilies

**Figure 4:**
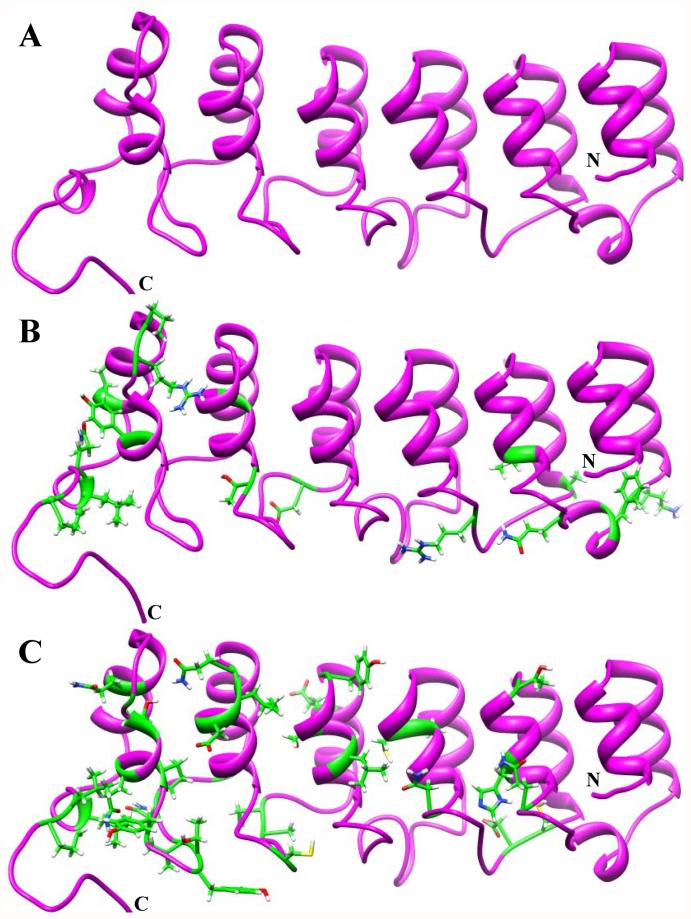
Type I and II functional divergence among IκB subfamily members. (A) Crystal structure of IκBα shown in ribbon representation (magenta). (B) Positions of type I sites based on the IκBα reference sequence. Type I sites are shown in stick representation (green). C. Positions of type II sites based on the IκBα reference sequence. Type II sites are shown in stick representation (green).

Gene duplications provide a means to develop novel biological functions, and changes in protein function may then cause different functional constraints on the subsequent evolution of the duplicated genes. Generally, the functional divergence of a protein family occurs after major evolutionary events such as speciation or gene duplication [63]. In order to estimate the relationship between gene evolution and the functional divergence of the IκB protein family, we conducted pairwise functional divergence analysis between the IκB genes by using DIVERGE 2.0 [48], [49]. In this type I functional analysis, we considered only the C-terminal ANK repeat region from 10 IκB subfamilies, which were consequently subjected to *a posteriori* analysis. Since the number of sequences per subfamily was quite large, we grouped a minimum of 4 sequences in each clade by both subfamily and taxonomic class, as required by DIVERGE. Using MSA (with a minimum of 4 sequences per subfamily for a total of 51 sequences), maximum likelihood tree topology, and the IκBα crystal structure, we determined the evolutionary rates of functional divergence of the IκBs using DIVERGE. The coefficient of evolutionary functional divergence (*θ*), its standard error, and the maximum likelihood ratio test (LRT) were determined for each pairwise comparison (Table 2). These 10 subfamily groups resulted in 45 pairwise group comparisons, of which 13 comparisons demonstrated statistically significant divergence (values shaded in gray in Table 2; LRT, p<0.05). Additionally, the type I functional divergence analysis showed medium to high *θ_I_* values between all IκB pairwise comparisons, except IκBβ/NF-κB1 (Table 2). The *θ_I_* values were >0 and statistically significant at the 1% level in accordance to LRT, thereby providing solid evidence of type I functional divergence between the IκB subfamilies. In order to identify the amino acid sites that might be involved in the functional divergence of the IκB family members, the significant values of *θ_I_* were compared using *a posteriori* probability analysis with a suitable cut-off value. A site that showed *θ_I_*>0.85 was thought to be a potential type I site. A total of 18 potential type I sites were identified in all pairwise comparisons. All of the predicted functional amino acid sites were found to be clustered among all of the ARDs of the IκBα subfamily member (Figures 1, 4A and B). We provided the residue positions based on the human IκBα reference sequence. Furthermore, the results show that the amino acid residues that are critical for functional divergence are located predominantly in the finger loop regions, but a few are also present in the helical region (Figures 1, 4A and B). It is noteworthy that the IκB finger loop regions mediate important interactions with different NF-κB subunits, thereby modulating the transcriptional process.

**Table 2:** Type I evolutionary functional divergence between IκB subfamilies based on taxonomic class.

IκB proteins	Alpha	Beta	Cactus	Epsilon	Bcl3	NF-κB1	NF-κB2	IκBNS	Zeta	Relish
**Alpha**	*******	0.2096±0.1288	0.2712±0.1260	0.2723±0.1196	0.3784±0.1269	0.2038±0.1183	0.2256±0.1118	0.5605±0.1029	0.4630±0.1240	0.5740±0.1217
**Beta**	1.6718	*******	0.1929±0.1545	0.2277±0.1473	0.2651±0.1802	−0.3538±0.0880	0.1694±0.1298	0.5392±0.1617	0.2807±0.1411	0.2993±0.1691
**Cactus**	3.0516	0.9261	*******	0.1557±0.1164	0.0026±0.1601	0.0701±0.1006	0.2521±0.1102	0.0961±0.1222	0.1879±0.1226	0.2582±0.1270
**Epsilon**	3.4632	1.5622	1.2081	*******	0.1516±0.1376	0.0276±0.0949	0.1359±0.1130	0.2667±0.1469	0.4181±0.1382	0.4485±0.1397
**Bcl3**	**5.2726**	1.2543	0.0001	0.7261	*******	0.4503±0.2086	0.2870±0.1866	0.6369±0.1578	0.4095±0.1907	0.4946±0.2788
**NF-κB1**	1.9907	ND	0.3057	0.0470	2.3783	*******	0.1029±0.1217	0.6285±0.1366	0.5021±0.1501	0.3961±0.1840
**NF-κB2**	2.9522	1.0735	**4.0838**	0.9039	1.2016	0.4066	*******	0.3337±0.1252	0.3395±0.1265	0.2882±0.1602
**IκBNS**	**17.7244**	**6.5346**	0.3513	1.9142	**7.1818**	**11.6496**	3.9172	*******	0.5062±0.2747	0.4803±0.1561
**Zeta**	**10.5010**	3.0359	1.7093	**7.8126**	2.3414	**7.9464**	**5.5268**	1.6882	*******	0.6957±0.1685
**Relish**										

Type I (θ_I_) functional divergence and its respective standard error are shown above the diagonal (upper right). Functional divergence values are represented by 1>θ>0.5. Below the diagonal (lower left) are the LRT values for testing the null hypothesis of θ = 0. The bolded LRT values significantly reject the null hypothesis (p≤0.05).

Amino acid residues with radical biochemical changes between the IκB subfamilies were identified via type II functional analysis. We did not perform IκB pairwise comparisons among all IκB subfamilies but performed pairwise comparisons within 8 vertebrate IκB subfamilies. We grouped a minimum of 4 sequences in each clade by both subfamily and taxonomic class (1 or 2 representative sequences for a total of 41 sequences), as required by DIVERGE. In contrast to the significant estimates of *θ_I_*, no evidence for type II functional divergence was observed (data not shown) between any of the pairwise groups with extremely small *θ_II_* values. Notably, most of the residues received very low scores, indicating that only a small portion of the amino acid residues have been involved in this type of functional divergence. Although there was no clear evidence for type II functional divergence (p>0.05), we performed a further analysis to determine whether any potential sites show evidence for type II functional divergence. We supposed that if the *a posteriori* ratio test value of an amino acid site was >17, then it could be considered as a potential type II site. Thus, 33 amino acid sites show a typical shift of amino acid properties at conserved residues (Figures 1 and 4C and Table 3). Most of these sites were distributed throughout the finger loop regions. The largest number of radical changes identified in type II functional analysis was found in the comparison between IκBα/IκBε, IκBα/IκBNS, and IκBα/IκBζ. The highest site-specific posterior probabilities were identified in the IκBα/IκBζ and IκBα/IκBNS pairwise comparisons, suggesting that the binding specificity toward NF-κB subunits/partners may be different between cytoplasmic and nuclear IκB proteins. The results from the analysis of type I and type II functional divergence (Tables 2 and 3) suggested that the IκB genes should be significantly functionally divergent, due to the differences in their evolutionary rates and amino acid properties at specific sites. Hence, the functional divergence of these proteins possibly reflects the existence of long-term selective pressure.

**Table 3:** Overview of the amino acid changes in the 33 predicted sites of type II functional divergence.

Residue position (Human IκBα)	Amino acid change	Property change
40* [635]#	Q → K	Hydrophilic/+
44 [639]	H → Q	+/Hydrophilic
49 [644]	T → A	Hydrophilic/Hydrophobic
63 [660]	C → A	Hydrophilic/Hydrophobic
64 [661]	D → Q	−/Hydrophilic
73 [670]	N → R	Hydrophilic/+
79 [676]	A → C	Hydrophobic/Hydrophilic
80 [677]	C → A	Hydrophilic/Hydrophobic
117 [785]	L → C	Hydrophobic/Hydrophilic
119 [787]	S → V	Hydrophilic/Hydrophobic
122 [790]	G → H	Hydrophilic/+
123 [791]	Y → N	Hydrophilic/Hydrophilic
128 [823]	E → K	−/+
138 [833]	N → E	Hydrophilic/−
139 [834]	A → S	Hydrophobic/Hydrophilic
143 [859]	C → K	Hydrophilic/+
154 [870]	D → Q	−/Hydrophilic
155 [871]	L → E	Hydrophobic/−
156 [872]	Q → A	Hydrophilic/Hydrophobic
170 [891]	D → F	−/Hydrophobic
172 [893]	N → D	Hydrophilic/−
174 [895]	V → K	Hydrophobic/+
175 [896]	T → A	Hydrophilic/Hydrophobic
176 [897]	Y → H	Hydrophilic/+
179 [900]	Y → N	Hydrophilic/Hydrophilic
181 [902]	P → A	Hydrophilic/Hydrophobic
183 [904]	Q → H	Hydrophilic/+
185 [906]	T → A	Hydrophilic/Hydrophobic
187 [912]	G → R	Hydrophilic/+
194 [919]	Q → V	Hydrophilic/Hydrophobic
205 [930]	L → S	Hydrophobic/Hydrophilic
208 [933]	L → N	Hydrophobic/Hydrophilic
209 [934]	P → L	Hydrophilic/Hydrophobic

Sites of radical change were detected in pairwise type II functional analysis of vertebrate IκB taxonomic groups. The residue positions are provided based on the human IκBα reference sequence. The amino acid changes between subfamilies along with their property changes are provided.

+ represents positively charged amino acids; − represents negatively charged amino acids;

* represents the residue position based on the human IκBα reference sequence;

# represents the residue position in the full vertebrate IκB alignment.

### Selective pressure at amino acid sites in the IκB family members

In order to test for the presence of positive selection at individual amino acid codons, we used the Selecton server [50], [51]. We estimated the number of synonymous (Ks) and non-synonymous (Ka) nucleotide substitutions per site for all of the IκB subfamily members. Likelihood rate tests were performed between model M8 (positive selection enabled evolutionary model, beta + ω≥1) and M8a (null model with no positive selection, beta + ω = 1) on IκB sequences. The M8 evolutionary model showed that the overall Ka/Ks ratio was <1 for all IκB subfamily members, except IκBβ, IκBNS, Relish and NF-κB1, indicating that the IκB proteins have undergone strong purifying selection pressure (data not shown). Moreover, the M8a null model showed a statistical significance for all IκB subfamily members. The major site-specific positive selection amino acids were: IκBβ: 333S and 337Q; IκBNS: 8V; Relish: 270D, 473N, 480K, 485A, 487P, G506, 510H, 550A, 556R, and 575T; and NF-κB1: 435M, 477G, 482T, 488G, 493S, and 494A. Interestingly, the sites that underwent positive selection were located outside the N- and C-terminal ARD regions, thereby indicating that the IκB ARDs have undergone strong purifying selection, suggesting their importance for the function of IκB proteins.

In addition to the above-mentioned sequence and phylogenetic analyses of the IκB family, we conducted detailed structural studies of the IκB ARDs to identify the differences responsible for their functional divergence. Firstly, we analyzed ARD crystal structures and identified the inter-conformation relationships. Secondly, we modeled the IκB structures and identified their domain motions by utilizing an anisotropic network model (ANM).

### PCA and ANM analysis of solved ARD protein structures

**Figure 5:**
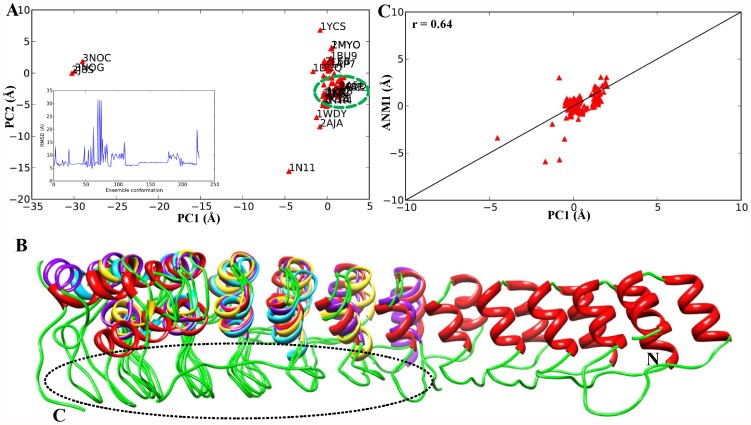
ARD structural analysis. (A) Projection of 227 ARD structures onto PC1 and PC2, represented as red triangles. The structures are separated into 4 distributions, and the NMR structures are labeled with their corresponding PDB IDs. The green dotted circle represents the cluster within which the IκBs are distributed. The inset shows the RMSD values of the 227 conformations, ranging from 4 to 30 Å. (B) Structural variations observed in the 4 distributions are illustrated using the selected structures (1N11, 1YCS, 1K1A, and 3NOG) labeled in (A). The 1N11, 1YCS, 1K1A, and 3NOG structures are in red, blue, purple and yellow, respectively. The finger loop regions are shown in green. (C) Projection of the 227 structures onto the PC1 and ANM1 directions.

Since our structural analysis focused on the ARDs of IκBs, we searched for similar types of ARD structures by using the RCSB Protein Data Bank (PDB). The complete list of 71 PDB identifiers can be found in the supporting information (Table S2). The objective of our PCA analysis of solved structures containing ARDs was to compare the functional variations in structures observed experimentally with those predicted from an ANM (physical theory and method) based on native contact topology. Figure 5A shows the projection of ARD structures onto the subspace spanned by the 2 principal axes PC1 and PC2. The points therein represent the distribution of 71 ARD structures. However, the ensemble represents 227 conformations because some of the structures in the dataset (Table S2) were represented by multiple NMR models. Each model was added to the ensemble as an individual conformation.

PC1 and PC2 were found to account for 62% of the total variance in structure. The projection of ARD structures onto PC1 and PC2 showed a clear separation of structures into 4 clusters according to the number of ANK repeats and function. Cluster 1 contained 3 structures, i.e., 3NOG, 3NOC, and 2J8S, which have 5 ANK repeats and function as signal transducers. Cluster 2 contained only a single structure, 1N11, which has 12 ANK repeats and acts as an ion transporter. Similarly, cluster 4 contained only a single structure, 1YCS, which has 4 ANK repeats and acts as a cell cycle regulator. However, cluster 3 contained 66 structures (including IκB protein structures), which have 6–11 ANK repeats and function as either transcriptional initiators or regulators. It should be noted that all of the IκB family proteins exhibited similar conformations with an RMSD of ∼5 Å, whereas the other ARD structures showed conformational changes with an RMSD ranging from 4 to 30 Å while binding to other proteins, as shown in the inset of Figure 5A.

The structural variations among the 4 clusters (PDB IDs: 1N11, 1YCS, 1K1A, and 3NOG) are shown in Figure 5B. Major conformational variations are clearly observed in the finger loop regions (Figure 5B, marked with black dotted circles), which are primarily responsible for the functional divergence among the ARD proteins. The correlations between the lowest 3 ANM modes for the Bcl3 structure (representing IκBs) and the top-ranking 3 PC directions are listed in Table 4. Among them, ANM1 had a correlation of 0.22 with PC1. The projections of the ensemble of structures onto ANM1 and PC1 yielded a correlation of 0.64 (Figure 5C). This correspondence underscores the robustness of low-frequency modes and demonstrates the functional importance of the ARD in Bcl3 (and IκBs), among other ARD functions. Overall, these results show that even though these ARD domains are from diverged families, they form the same folded structure, and the variations are mainly observed in the number of ANK repeats and in the finger loop regions, which are primarily responsible for the functional divergence among ARD-containing proteins.

**Table 4:** Overlap between PCA and ANM modes.

PCA modes (fractional contribution)	ANM modes			
Ankyrin repeat domain-containing structures	Principle components	ANM1	ANM2	ANM3
	PC1 (0.38)	0.22	0.01	0.12
	PC2 (0.24)	0.05	0.07	0.01
	PC3 (0.16)	0.13	0.15	0.01

### Homology modeling of IκB proteins

To date, the crystal structures of 2 cytoplasmic IκBs (IκBα and IκBβ) and 1 nuclear IκB protein (Bcl3) have been solved [60], [62], [64]. We built the remaining IκB protein structures (IκBε, IκBζ, IκBNS, Cactus, Relish, NF-κB1, and NF-κB2) using comparative modeling. On the basis of sequence identity, the nuclear IκB protein Bcl3 is a suitable template for IκBNS, NF-κB1, NF-κB2, and IκBζ 3D modeling, whereas for IκBε, both IκBβ and Bcl3 can serve as templates. Furthermore, IκBα (1IKN) serves as a suitable template for Cactus, while another ARD, 1N11, serves as a suitable template for Relish. During target-template alignments of IκBNS, IκBζ, Cactus and Relish, we identified insertions (20, 27, 31, and 14 amino acids in length, respectively), which did not align with the template; however, we did not delete these insertion regions when building the models (Figure S2). Model evaluation involves the analysis of the geometry, stereochemistry and energy distribution of the final models. The evaluation listed in Table 5 indicated that all of the models were of high quality in terms of overall packing. The final structure of Cactus contained 6 ANK repeats, whereas those of IκBNS, IκBε, IκBζ, Relish, NF-κB1 and NF-κB2 contained 7 ANK repeats. All of the predicted structures were in agreement with the secondary structure predictions made using PSIPRED [65]. In order to identify the domain motion for each IκB member, 6 IκB models (IκBα, IκBβ, IκBε, IκBNS, IκBζ and Bcl3) were subjected to ANM analysis.

**Table 5:** Model evaluation.

Protein	ProQ_LG/MX	MetaMQAP_GDT/RMSD	DOPE Score	Z-Score
IκBε	3.518/0.242	48.092/4.089	−22734.0566	−7.0322
IκBNS	5.228/0.325	59.684/2.725	−25000.9805	−9.9145
IκBζ	4.617/0.312	49.060/4.155	−25130.0215	−7.0121
Relish	4.050/0.220	51.914/4.056	−23081.6074	−7.9487
NF-κB1	4.832/0.322	50.800/3.693	−24206.1484	−9.7448
NF-κB2	5.029/0.299	55.449/2.885	−23566.7383	−9.3233
Cactus	3.669/0.293	47.826/4.019	−21998.7891	−6.9505

The displayed IκB models are reliable in terms of overall packing. ProQ_LG: >1.5, fairly good; >2.5, very good; >4, extremely good. ProQ_MX: >0.l, fairly good; >0.5, very good; >0.8, extremely good. MetaMQAP_GDT/RMSD: an ideal model has a GDT score of >59 and an RMSD of ∼2.0 Å. Low DOPE scores indicate best model.

### Conformational flexibility of the IκB proteins

The protein peptide backbone and side chains exist in a state of constant motion; these internal local movements are quite fast, i.e., in the femto- to pico-second time scales [66]. In a previous study, we conducted molecular dynamics (MD) simulations (15 ns) for most of the IκB proteins [2]. We found that the proteins reach equilibrium after 3 or 4 ns and remain stable thereafter, without undergoing any major conformational changes. We then extended our MD simulations to 50 ns and noticed that the proteins reached an equilibrium state after 4 ns and remained stable until 40 ns, after which they entered a metastable state and then reached a second equilibrium state after 45 ns (manuscript in preparation, data not shown). These results clearly suggest that IκB proteins require a slower conformational transition, involving medium- to large-scale motions (on a microsecond scale). In order to identify the mobility occurring within the ANK repeat, we utilized ANM analysis. The rationale for using NMA, and, in particular, of using elastic network models (ENMs) over traditional methods such as MD, was based on its fast computational time and ability to predict large-scale fluctuations and global motion. The IκB protein family and the finger loop regions within the ARDs play an important role in binding specificity. The ANM was expected to provide significant information about these regions.

### Fluctuations predicted by the ANM correlate well with the experimental data

**Figure 6:**
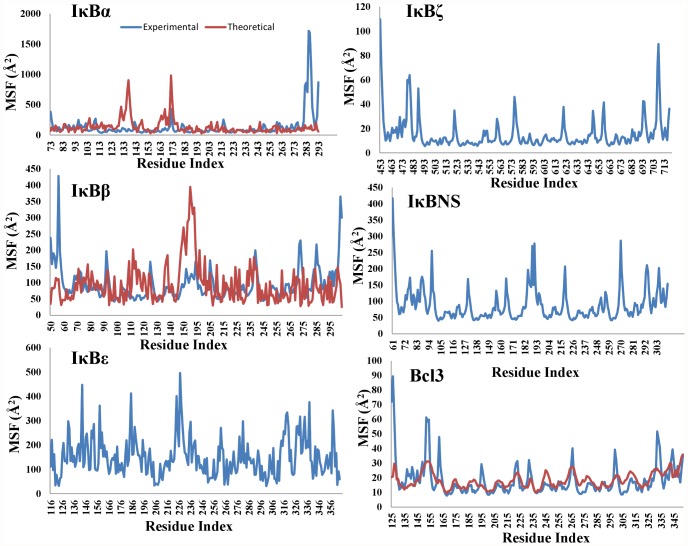
Calculated and experimental B-factors. The calculated and experimental B-factors for known IκB crystal structures such as IκBα, IκBβ, and Bcl3 are shown as a function of residue number, whereas for IκBε, IκBζ, and IκBNS, only the calculated B-factors are shown as a function of residue number.

Among the 6 IκB structures, 3 were taken from the crystallographic coordinates. We analyzed the functional motion of these structures using the ANM. Importantly, the crystal structures can reproduce experimental data, e.g., temperature, and also provide cooperative, global protein motions at low frequencies. A comparison of the calculated (theoretical) and experimental fluctuations shown in Figure 6 indicates that there is qualitative agreement between the predicted mean square fluctuation and the experimental B-factors. This analysis shows that the ANM is successful in reproducing the experimental results. Apart from the crystal structures, we predicted the theoretical fluctuations for the modeled proteins. In the fluctuation plots, the maxima indicate the areas of the protein structure with high flexibility, whereas the minima correspond to regions with restricted flexibility. The highly flexible regions generally coincided with the positions of ANK1, ANK6, or ANK7 and the finger loop regions, whereas the low mobility areas correspond to residues in the helical segment.

### Direction of motions

We analyzed the direction of motion for the first 3 low-frequency modes of each IκB protein. The mobile regions identified for IκB-α, -β, -ε, and –ζ, and the Bcl3 proteins were relatively similar to the flexible residues identified from MD simulation studies [2]. In most cases, domain movement correlated well with the experimental observations.

**Figure 7:**
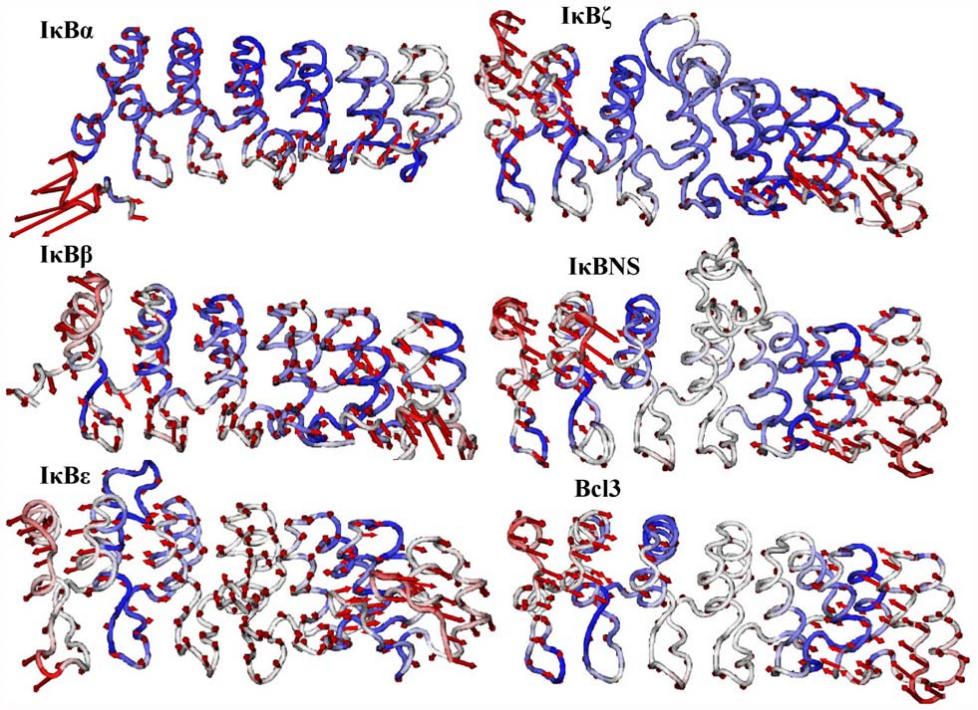
Lowest frequency mode 1 obtained for IκB proteins using ANM. The lowest frequency mode 1 results are shown from top to bottom. Only the Cα atoms are shown for clarity. The atoms in red indicate large fluctuations, whereas the blue color corresponds to small fluctuations. The magnitude and direction of the displacement for mode 1 are represented by the red arrows.

**Figure 8:**
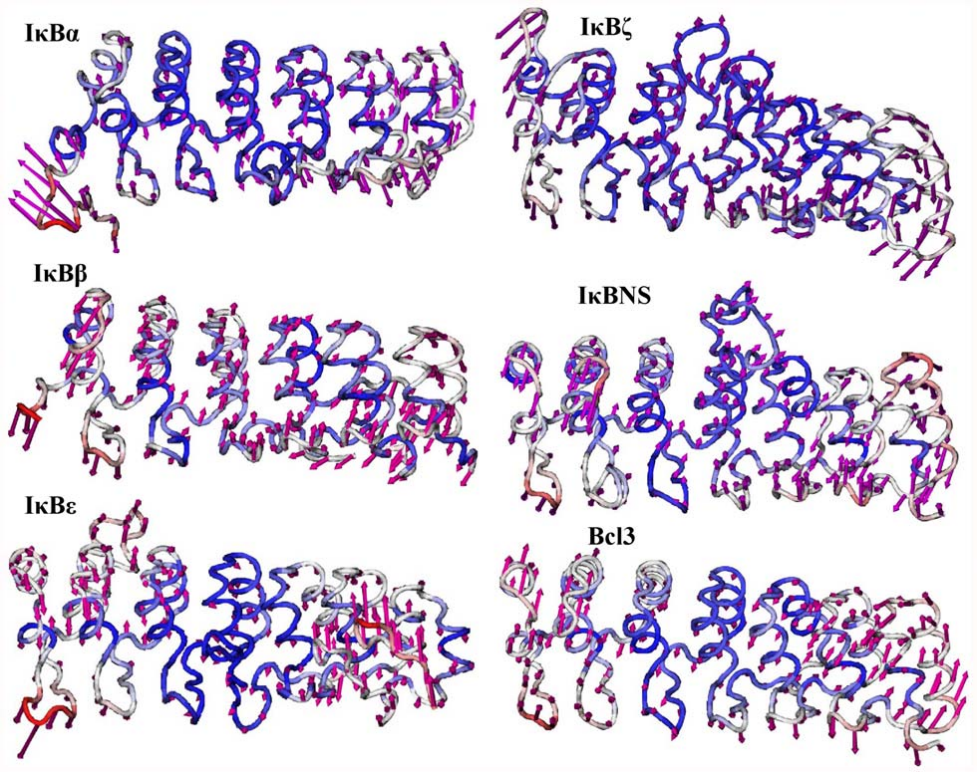
Lowest frequency mode 2 obtained for IκB proteins using ANM. The lowest frequency mode 2 results are shown from top to bottom. Only the Cα atoms are shown for clarity. The atoms in red indicate large fluctuations, whereas blue color corresponds to small fluctuations. The magnitude and direction of the displacement for mode 2 are represented by the magenta arrows.

**Figure 9:**
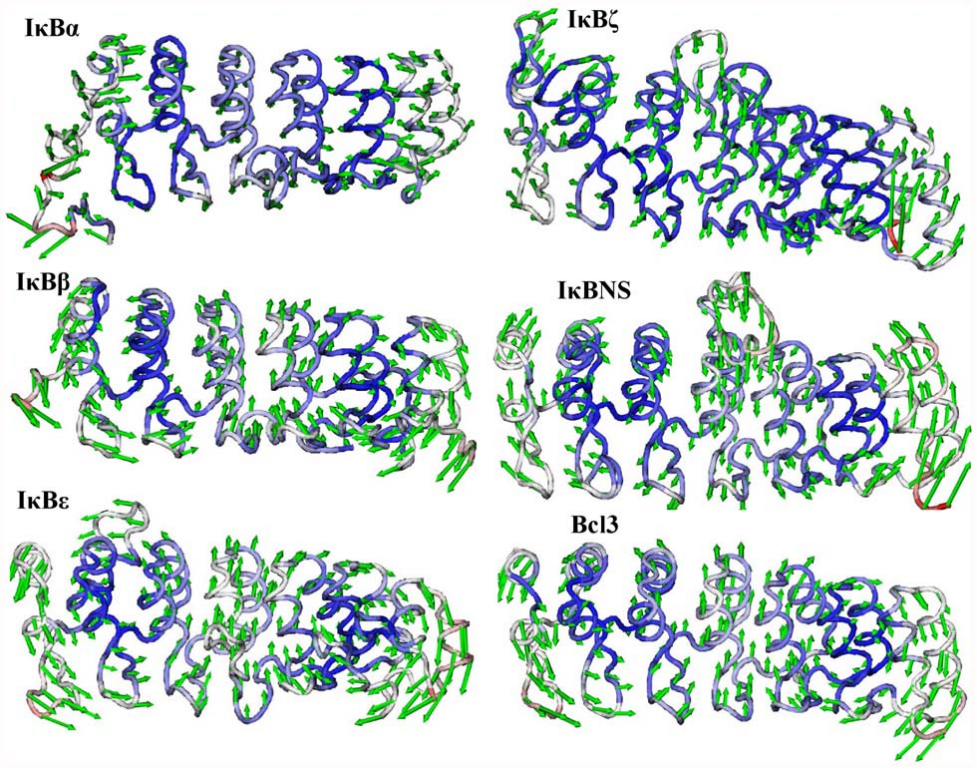
Lowest frequency mode 3 obtained for IκB proteins using ANM. The lowest frequency mode 3 results are shown from top to bottom. Only the Cα atoms are shown for clarity. The atoms in red indicate large fluctuations, whereas the blue color corresponds to small fluctuations. The magnitude and direction of the displacement for mode 3 are represented by the green arrows.

In the case of IκBα, large domain movement was observed at the C-terminal end, which encompasses the PEST motif. This region plays a major role in disrupting the p50/p65-DNA complex and retaining the IκBα-p50/p65 complex in the cytoplasm [58]. At the N-terminus, we noticed a few flexible regions in areas that are important for masking the NLS of the p50 and p65 subunits [60], [61]. Apart from these regions, fluctuating residues were also observed in the third finger loop region, the function of which remains unknown (Figures 7, 8, and 9 and Figure S3). The domain motion identified in IκBβ was similar to that in IκBα (Figures 7, 8 and 9 and Figure S3). The function of the N-terminal region is to mask the NLS of the p65/p65 homodimer [62]. Although the crystal structure of IκBβ bound to the p65/p65 homodimer is available, and is largely similar to that of IκBα, the high-mobility PEST region projects away from the protein-protein surface, clearly indicating that it has a different function. Recent biochemical studies have shown that the S313 and S315 residues in the PEST motif undergo phosphorylation and interact functionally with the cytoplasmic inhibitor c-Rel [67]. Apart from these, the fifth finger loop region also showed mobility; however, this region does not participate in the interface. Among the cytoplasmic IκBs, IκBε contains 7 ANK repeats, in common with nuclear IκBs; however, its dynamic motion was similar to that of the above-described cytoplasmic IκBs (Figures 7, 8 and 9 and Figure S3). The function of the N-terminal region is to mask the NLS of the p50 and p65 subunits, while the C-terminal region has high mobility and contains negatively charged amino acids that probably play a similar role to those in IκBα, with delayed kinetics [2], [68].

IκBNS exhibited motion at the N-terminus and in the C-terminal finger loop regions (Figures 7, 8, and 9 and Figure S3), which play an important role in interacting with the p50/p50 dimerization interface [2]. Unexpectedly, we noted loop movement within ANK4; further biochemical studies are required to clarify the function of this region. Among the nuclear IκBs, IκBζ possesses numerous functions, but the motion predicted by the current study was in the N-terminal and C-terminal regions. The N-terminus contributes to the binding of the p50 and p65 subunits, whereas the C-terminal regions are known to be involved in binding the p50/p50 homodimer [2]. Moreover, the theoretical B-factor identified 2 additional regions exhibiting motion in the fourth and fifth finger loops (Figure 6). Of these, the fourth finger loop region is responsible for its interaction with the p50 and p65 subunits, whereas the fifth finger loop region contributes to its interaction with the p50/p50 homodimer. In the case of Bcl3, the function of the identified N-terminal cluster remains largely unknown, whereas the C-terminal mobility region is important for its binding with p50 subunits and the subsequent removal of the Bcl3-p50/p50 complex from DNA (Figures 7, 8, and 9 and Figure S3), resulting in the binding of an active NF-κB dimer to DNA [2]. Generally, nuclear IκBs are produced along with the products of the primary response genes.

NMA showed a similar pattern of domain motion for all IκB family members, although the residues involved in the motion of the domains are not evolutionarily conserved. However, we identified 2 amino acids (D73 and D75) in the IκBα N-terminal region whose corresponding positions were highly conserved among IκB members. In the case of cytoplasmic IκBs, these amino acid side chains point toward the NLS of the NF-κB subunits, thereby retaining the IκB/NF-κB complex in the cytoplasm [2], [6], [60], [61], [62]. However, in nuclear IκBs, these acidic residues point in the opposite direction, indicating that nuclear proteins do not have any functional influence over the NLS [2]. This clearly shows that conformational changes take place in these protein structures that keep the side chains of particular amino acids in an appropriate position, and are thus responsible for specific biological functions.

## Discussion

A wide variety of microbial components activate NF-κB, which plays an essential role in the optimal activation of the host immune system. The transcriptional activity of NF-κB is tightly regulated at multiple steps of the immune signaling pathways because its excessive activation is harmful to the host [16]. The activity of NF-κB is primarily controlled by a family of regulatory proteins called inhibitory IκB proteins. We have previously investigated the structural basis for the activation and inhibition of IκB proteins [2], [38]. However, in our current study, we performed a comprehensive bioinformatics analysis of the entire IκB family and identified their evolutionary, structural and functional relationships. Here, we have shown that the IκB family is much larger than previously anticipated [28] and it is composed of 11 members, namely, Relish, NF-κB1, NF-κB2, Cactus, IκBα, IκBβ, IκBγ, IκBε, IκBζ, IκBNS and Bcl3. Even though IκBγ is an IκB protein, we have not included it in our analysis since IκBγ corresponds to the C-terminal part of NF-κB1, which contains ANK repeats that play a potent role in auto-inhibition and is eliminated during maturation.

On the basis of our phylogenetic analysis, we identified Relish as the ancestor of IκB family members, which is in agreement with previous reports [8], [28]. Our phylogeny analysis organized the IκB subfamily members into 5 distinct clusters (Figure 2). In cluster 1, NF-κB1/NF-κB2 subclades were first separated from the ancient Relish subfamily by gene duplication. Relish, NF-κB1 and NF-κB2 genes contain 7 ANK repeats in their C-terminal regions and an RHD in their N-terminal regions. In addition to the identification of Relish as an early offshoot, the presence of both RHD and ARD suggests that the Relish genes may have diverged before the NF-κB genes as a result of an early gene duplication, which is in agreement with the findings of Huguet et al. [28]. IκBα and Cactus (*Drosophila* homolog) genes contain 6 ANK repeats representing the second cluster. Even though we can hypothesize that Cactus/IκBα represent paralogous genes from the phylogenetic tree, this is not possible since we were unable to identify a Cactus gene in vertebrates. Hence, it is clear that no vertebrate-specific gene duplication has given rise to a paralogous version of the IκBα gene, but the discovery of a new paralog is still possible. Clusters 3 and 4 represent the IκBε and IκBβ subfamilies; however, the relationship of these subtypes with other IκB family members remains unclear. Yet, their structures (the presence of 6–7 ANK repeats) and regulation are very close to those of IκBα; hence, these IκBs fall into the cytoplasmic IκB proteins. Cluster 5 is represented by nuclear IκB proteins such as Bcl3, IκBζ and IκBNS. Unlike Cactus/IκBα, vertebrate-specific gene duplication has given rise to the paralogous genes IκBNS and IκBζ.

Our phylogenetic analysis suggests that (a) the acquisition of the RHD in an unknown ancestor containing the ANK motif gave rise to the IκB genes (Relish, NF-κB1 and NF-κB2) and that (b) at a later evolutionary stage, a few unknown IκB genes lost their RHD and evolved into different IκB subfamilies such as Cactus, IκBα, β, ε, ζ, IκBNS and Bcl3. This is in line with previous studies demonstrating 2 models of evolution for the IκB proteins [28], [57]. The ANK domain appears to be more ancient than the RHD due to the presence of ANK repeat proteins in bacteria; hence, it is certain that this protein-protein interaction domain existed long before the development of the NF-κB pathway [57]. Considering all of these observations, we believe that the 8 IκB proteins in mammals arose due to the requirements of IκBs for different NF-κB/Rel complexes, different biological functions such as co-activators or inhibitors of NF-κB/Rel complexes, and differential tissue expression patterns. Overall, our phylogenetic data indicate that the IκB subfamily members evolved by several gene duplication events, divergence and acquisition or scission events in specific lineages, which is in agreement with the previous finding that large-scale gene duplications have occurred during chordate evolution [69], [70], [71], [72]. Thus, our phylogenetic analysis is conclusive about the relationships of the different IκB proteins.

Processes such as pseudogene formation [73], subfunctionalization [74], and neofunctionalization [75] after gene duplication may result in altered functional constraints between members of a gene family. In order to identify the functional divergence among the IκB subfamilies, we performed intensive type I and II functional divergence analyses (Figures 1 and 4). These analyses showed that type I rather than type II functional divergence is the main pattern for the functional divergence of the IκB subfamilies (Tables 2 and 3). In this study, the divergence of amino acid residues among the different IκB subfamilies provided us with an indication that the IκB genes may have diverse physiological functions and partner binding specificities, which is in agreement with the findings of several biochemical and structural studies [2], [6], [18], [19], [20], [21], [22], [23], [25], [26], [27], [38], [61], [62]. To further characterize the relationship between the site-specific evolution of amino acids and functional divergence, the potential amino acid sites related to positive selection and type I and II functional divergence were selected and mapped on to the reference IκBα structure as well as the sequence alignment. The analysis of type I and II functional divergence showed that nearly three-quarters of the functional divergence sites are distributed throughout the ARD finger loop regions, but a few are also present in the helical region, suggesting that IκB genes are functionally divergent and exhibit diverse binding specificities with NF-κB/Rel subunits (Figures 1 and 4). However, in the analysis of selective pressure for the IκB genes, site-specific positive selection amino acids were not identified in the functional ARD region but rather they were distributed in the outer regions of the ARDs, suggesting that the IκB proteins (ARDs) have undergone strong purifying selection and are functionally significant.

In addition to these evolutionary and functional analyses, we were also interested in exploring the differences accountable for the functional divergence in IκBs; hence, structural studies of the IκB ARDs were conducted. Our PCA analysis of ARD structures (Figure 5) correlated well with the structural superimposition studies demonstrating that even though the structures are similar, major variations took place primarily in the number of ANK repeats and in the finger loop regions, which are primarily responsible for the observed functional divergence among IκB family members. In order to check for the functionally relevant motion and directionality of the IκBs, we utilized NMA. NMA has been successfully used in determining domain motions and their collective nature for various types of proteins, including hexokinases [76], RNA polymerases [77], lysozymes [78], citrate synthetase [79], adenylate kinase [80], and icosahedral virus capsid proteins [81]. Our NMA analysis identified mobile regions that are located on the loops connecting the regular secondary structural elements (Figures 7, 8, and 9 and Figure S3). Since the loops have a higher degree of freedom than the highly packed regions (i.e., helices), they are usually anticipated to have high mobility. Nevertheless, the slowest modes identified from our ENMs were highly specific; only certain loops showed large domain movement or motion, which suggests that these mobile loops may have functional roles in the interaction with different NF-κB/Rel subunits. Indeed, a few residues identified in the loop region contribute to the binding interface with NF-κB dimers, which are in agreement with previous biochemical and structural studies [2], [6], [18], [19], [20], [21], [22], [23], [25], [26], [27], [38], [61], [62]. Further mutational or deletion experiments are required to identify which specific residue sites on the loops are essential for the functional specificity exhibited.

Moreover, it should be noted that although we did not observe any conserved residues contributing to domain motion across all of the IκB genes, we noticed some conspicuous conservation among cytoplasmic and nuclear IκBs. These conserved residues exhibiting domain motion are probably the driving force for binding with the same NF-κB units that were observed in a few cytoplasmic and nuclear IκBs. However, different IκB homologs have different degradation and resynthesis rates and thus regulate NF-κB activation with unique kinetics after a particular stimulus [1], [82], [83].

In conclusion, this study may help to understand the phylogeny and structural and functional divergence of IκB family members more clearly. Gene duplications, gene divergence, acquisition, or scission events of certain domains that depend on the evolutionary rate and purifying selection are the primary evolutionary forces involved in the generation of the IκB family. Structural and functional analyses support the hypothesis that the IκB proteins have evolved distinctive functional properties due to the variations in their ANK repeats, high fluctuations observed in their ARD finger loop regions, and the divergence of amino acid sequences among different IκB subfamilies.

Our current study represents the first thorough examination of the evolution, structure, and function of the IκB family. Our evolutionary analysis proposes that all IκB subfamilies have diverged and been duplicated from a single, common, ancestral RHD-containing ANK gene. We utilized 3D modeling to demonstrate that all the IκB proteins share similar structural folds and are likely to retain similar functions. However, in our structural and functional analyses, the divergence of amino acid sequences among different IκB subfamilies, variation in the number of ANK repeats, and higher flexibility in the finger loop regions provided us with an indication that the IκB family genes may have diverse physiological functions and distinct binding affinities for different NF-κB/Rel partners. Overall, our study demonstrates the utility of a multidisciplinary approach that combines insights from the evolutionary origins of IκBs with computational methods, leading to the prediction of structural and functional divergence that can be tested at the molecular level.

## Supporting Information

Figure S1 **Domain organization and structural superimposition of IκB family members.** (A) Domain organizations of the IκB subfamily members are shown. The number of amino acids in each protein is indicated on the right. NES, nuclear export sequence; NLS, nuclear localization signal; TAD, transactivation domain; DD, death domain; CC, coiled-coil domain; RHD, Rel-containing homology domain; and GRR, glycine-rich repeat. (B) Structural superimposition of typical IκB proteins and (C) IκB-like domain containing proteins. Major variations are shown by black stars in the ribbon representation of the IκB subfamily members. IκB proteins are colored as follows: IκBα – magenta; IκBβ – orange; IκBε – purple; IκBζ – forest green; IκBNS – yellow; Bcl3 – red; Cactus – cornflower blue; Relish – green; NF-κB1 – dark blue; and NF-κB2 – cyan.
(TIF)

Figure S2 **Comparative models of IκB proteins.** Crystallographic structures of IκB proteins such as IκBα, IκBβ and Bcl3, are shown in ribbon representation. Other IκB proteins such as IκBε, IκBζ, IκBNS, Relish, Cactus, NF-κB1 and NF-κB2, are modeled structures. IκB proteins are colored as follows: IκBα – magenta; IκBβ – orange; IκBε – purple; IκBζ – forest green; IκBNS – yellow; Bcl3 – red; Cactus – cornflower blue; Relish – green; NF-κB1 – dark blue; and NF-κB2 – cyan. The insertion regions in the modeled and crystal structures are highlighted with dotted circles.
(TIF)

Figure S3 **Fluctuation profiles of the 3 lowest frequency normal modes for the IκB subfamilies.** The 3 lowest frequency normal modes obtained for the IκB proteins using ANM are shown. Fluctuation profiles have been shown as a function of residue number corresponding to the 3 lowest frequency modes. Mode 1 is shown in blue, mode 2 in red, and mode 3 in green.
(TIF)

Table S1 IκB Homologs (n = 340) Used for Phylogenetic Analysis. This table lists the molecular features of all 340 IκB homologs identified in public databases that were utilized for IκB phylogenetic tree reconstructions (all, vertebrate and invertebrate).
(XLS)

Table S2 **PDB structures.** The PDB structures that were utilized in our PCA analysis were resolved at 1.2 Å or higher. Bcl3 (PDB ID: 1K1A) was used as the reference structure. The numbers next to the PDB IDs indicate the number of individual NMR ensembles.
(DOC)

## References

[1] Hoffmann A, Baltimore D (2006) Circuitry of nuclear factor kappaB signaling. Immunol Rev 210: 171–186. 16623771 10.1111/j.0105-2896.2006.00375.x

[2] Manavalan B, Basith S, Choi YM, Lee G, Choi S (2010) Structure-function relationship of cytoplasmic and nuclear IkappaB proteins: an in silico analysis. PLoS One 5: e15782. 21203422 10.1371/journal.pone.0015782 PMC3009747

[3] Baeuerle PA, Baltimore D (1988) Activation of DNA-binding activity in an apparently cytoplasmic precursor of the NF-kappa B transcription factor. Cell 53: 211–217. 3129195 10.1016/0092-8674(88)90382-0

[4] Li J, Mahajan A, Tsai MD (2006) Ankyrin repeat: a unique motif mediating protein-protein interactions. Biochemistry 45: 15168–15178. 17176038 10.1021/bi062188q

[5] Manavalan B, Basith S, Choi S (2012) IkBz. In: Choi S, editor. Encyclopedia of Signaling Molecules. 1 ed. New York: Springer. pp. 892–899.

[6] Yamauchi S, Ito H, Miyajima A (2010) IkappaBeta, a nuclear IkappaB protein, positively regulates the NF-kappaB-mediated expression of proinflammatory cytokines. Proc Natl Acad Sci U S A 107: 11924–11929. 20547855 10.1073/pnas.0913179107 PMC2900662

[7] Cheng JD, Ryseck RP, Attar RM, Dambach D, Bravo R (1998) Functional redundancy of the nuclear factor kappa B inhibitors I kappa B alpha and I kappa B beta. J Exp Med 188: 1055–1062. 9743524 10.1084/jem.188.6.1055 PMC2212550

[8] Dushay MS, Asling B, Hultmark D (1996) Origins of immunity: Relish, a compound Rel-like gene in the antibacterial defense of Drosophila. Proc Natl Acad Sci U S A 93: 10343–10347. 8816802 10.1073/pnas.93.19.10343 PMC38386

[9] Siebenlist U, Franzoso G, Brown K (1994) Structure, regulation and function of NF-kappa B. Annu Rev Cell Biol 10: 405–455. 7888182 10.1146/annurev.cb.10.110194.002201

[10] Inoue J, Kerr LD, Kakizuka A, Verma IM (1992) I kappa B gamma, a 70 kd protein identical to the C-terminal half of p110 NF-kappa B: a new member of the I kappa B family. Cell 68: 1109–1120. 1339305 10.1016/0092-8674(92)90082-n

[11] Belvin MP, Anderson KV (1996) A conserved signaling pathway: the Drosophila toll-dorsal pathway. Annu Rev Cell Dev Biol 12: 393–416. 8970732 10.1146/annurev.cellbio.12.1.393

[12] Krishnan J, Selvarajoo K, Tsuchiya M, Lee G, Choi S (2007) Toll-like receptor signal transduction. Exp Mol Med 39: 421–438. 17934330 10.1038/emm.2007.47

[13] Basith S, Manavalan B, Lee G, Kim SG, Choi S (2011) Toll-like receptor modulators: a patent review (2006–2010). Expert Opin Ther Pat 21: 927–944. 21406035 10.1517/13543776.2011.569494

[14] Manavalan B, Basith S, Choi S (2011) Similar Structures but Different Roles - An Updated Perspective on TLR Structures. Front Physiol 2: 41. 21845181 10.3389/fphys.2011.00041 PMC3146039

[15] Basith S, Manavalan B, Yoo TH, Kim SG, Choi S (2012) Roles of toll-like receptors in Cancer: a double-edged sword for defense and offense. Arch Pharm Res 35: 1297–1316. 22941474 10.1007/s12272-012-0802-7

[16] Yamamoto M, Takeda K (2008) Role of nuclear IkappaB proteins in the regulation of host immune responses. J Infect Chemother 14: 265–269. 18709529 10.1007/s10156-008-0619-y

[17] Huxford T, Ghosh G (2009) A structural guide to proteins of the NF-kappaB signaling module. Cold Spring Harb Perspect Biol 1: a000075. 20066103 10.1101/cshperspect.a000075 PMC2773632

[18] Rao P, Hayden MS, Long M, Scott ML, West AP, et al (2010) IkappaBbeta acts to inhibit and activate gene expression during the inflammatory response. Nature 466: 1115–1119. 20740013 10.1038/nature09283 PMC2946371

[19] Bours V, Franzoso G, Azarenko V, Park S, Kanno T, et al (1993) The oncoprotein Bcl-3 directly transactivates through kappa B motifs via association with DNA-binding p50B homodimers. Cell 72: 729–739. 8453667 10.1016/0092-8674(93)90401-b

[20] Carmody RJ, Ruan Q, Palmer S, Hilliard B, Chen YH (2007) Negative regulation of toll-like receptor signaling by NF-kappaB p50 ubiquitination blockade. Science 317: 675–678. 17673665 10.1126/science.1142953

[21] Fiorini E, Schmitz I, Marissen WE, Osborn SL, Touma M, et al (2002) Peptide-induced negative selection of thymocytes activates transcription of an NF-kappa B inhibitor. Mol Cell 9: 637–648. 11931770 10.1016/s1097-2765(02)00469-0

[22] Kuwata H, Matsumoto M, Atarashi K, Morishita H, Hirotani T, et al (2006) IkappaBNS inhibits induction of a subset of Toll-like receptor-dependent genes and limits inflammation. Immunity 24: 41–51. 16413922 10.1016/j.immuni.2005.11.004

[23] Motoyama M, Yamazaki S, Eto-Kimura A, Takeshige K, Muta T (2005) Positive and negative regulation of nuclear factor-kappaB-mediated transcription by IkappaB-zeta, an inducible nuclear protein. J Biol Chem 280: 7444–7451. 15618216 10.1074/jbc.M412738200

[24] Totzke G, Essmann F, Pohlmann S, Lindenblatt C, Janicke RU, et al (2006) A novel member of the IkappaB family, human IkappaB-zeta, inhibits transactivation of p65 and its DNA binding. J Biol Chem 281: 12645–12654. 16513645 10.1074/jbc.M511956200

[25] Matsuo S, Yamazaki S, Takeshige K, Muta T (2007) Crucial roles of binding sites for NF-kappaB and C/EBPs in IkappaB-zeta-mediated transcriptional activation. Biochem J 405: 605–615. 17447895 10.1042/BJ20061797 PMC2267307

[26] Trinh DV, Zhu N, Farhang G, Kim BJ, Huxford T (2008) The nuclear I kappaB protein I kappaB zeta specifically binds NF-kappaB p50 homodimers and forms a ternary complex on kappaB DNA. J Mol Biol 379: 122–135. 18436238 10.1016/j.jmb.2008.03.060

[27] Wu Z, Zhang X, Yang J, Wu G, Zhang Y, et al (2009) Nuclear protein IkappaB-zeta inhibits the activity of STAT3. Biochem Biophys Res Commun 387: 348–352. 19595668 10.1016/j.bbrc.2009.07.023

[28] Huguet C, Crepieux P, Laudet V (1997) Rel/NF-kappa B transcription factors and I kappa B inhibitors: evolution from a unique common ancestor. Oncogene 15: 2965–2974. 9416840 10.1038/sj.onc.1201471

[29] Altschul SF, Madden TL, Schaffer AA, Zhang J, Zhang Z, et al (1997) Gapped BLAST and PSI-BLAST: a new generation of protein database search programs. Nucleic Acids Res 25: 3389–3402. 9254694 10.1093/nar/25.17.3389 PMC146917

[30] Benson DA, Karsch-Mizrachi I, Lipman DJ, Ostell J, Wheeler DL (2004) GenBank: update. Nucleic Acids Res 32: D23–26. 14681350 10.1093/nar/gkh045 PMC308779

[31] Finn RD, Mistry J, Tate J, Coggill P, Heger A, et al (2008) The Pfam protein families database. Nucleic Acids Res 38: D211–222. 10.1093/nar/gkp985 PMC2808889 19920124

[32] McDowall J, Hunter S (2011) InterPro protein classification. Methods Mol Biol 694: 37–47. 21082426 10.1007/978-1-60761-977-2_3

[33] Olson SA (2002) EMBOSS opens up sequence analysis. European Molecular Biology Open Software Suite. Brief Bioinform 3: 87–91. 12002227 10.1093/bib/3.1.87

[34] Rice P, Longden I, Bleasby A (2000) EMBOSS: the European Molecular Biology Open Software Suite. Trends Genet 16: 276–277. 10827456 10.1016/s0168-9525(00)02024-2

[35] Katoh K, Misawa K, Kuma K, Miyata T (2002) MAFFT: a novel method for rapid multiple sequence alignment based on fast Fourier transform. Nucleic Acids Res 30: 3059–3066. 12136088 10.1093/nar/gkf436 PMC135756

[36] Waterhouse AM, Procter JB, Martin DM, Clamp M, Barton GJ (2009) Jalview Version 2–a multiple sequence alignment editor and analysis workbench. Bioinformatics 25: 1189–1191. 19151095 10.1093/bioinformatics/btp033 PMC2672624

[37] Ronquist F, Huelsenbeck JP (2003) MrBayes 3: Bayesian phylogenetic inference under mixed models. Bioinformatics 19: 1572–1574. 12912839 10.1093/bioinformatics/btg180

[38] Manavalan B, Govindaraj R, Lee G, Choi S (2011) Molecular modeling-based evaluation of dual function of IkappaBzeta ankyrin repeat domain in toll-like receptor signaling. J Mol Recognit 24: 597–607. 21472811 10.1002/jmr.1085

[39] Basith S, Manavalan B, Govindaraj RG, Choi S (2011) In silico approach to inhibition of signaling pathways of Toll-like receptors 2 and 4 by ST2L. PLoS One 6: e23989. 21897866 10.1371/journal.pone.0023989 PMC3163686

[40] Manavalan B, Murugapiran SK, Lee G, Choi S (2010) Molecular modeling of the reductase domain to elucidate the reaction mechanism of reduction of peptidyl thioester into its corresponding alcohol in non-ribosomal peptide synthetases. BMC Struct Biol 10: 1. 20067617 10.1186/1472-6807-10-1 PMC2835699

[41] Edgar RC (2004) MUSCLE: multiple sequence alignment with high accuracy and high throughput. Nucleic Acids Res 32: 1792–1797. 15034147 10.1093/nar/gkh340 PMC390337

[42] Eswar N, Webb B, Marti-Renom MA, Madhusudhan MS, Eramian D, et al (2006) Comparative protein structure modeling using Modeller. Curr Protoc Bioinformatics Chapter 5: Unit 5–6. 10.1002/0471250953.bi0506s15 PMC4186674 18428767

[43] Jo S, Kim T, Iyer VG, Im W (2008) CHARMM-GUI: a web-based graphical user interface for CHARMM. J Comput Chem 29: 1859–1865. 18351591 10.1002/jcc.20945

[44] Shen MY, Sali A (2006) Statistical potential for assessment and prediction of protein structures. Protein Sci 15: 2507–2524. 17075131 10.1110/ps.062416606 PMC2242414

[45] Wallner B, Fang H, Elofsson A (2003) Automatic consensus-based fold recognition using Pcons, ProQ, and Pmodeller. Proteins 53 Suppl 6: 534–541. 14579343 10.1002/prot.10536

[46] Pawlowski M, Gajda MJ, Matlak R, Bujnicki JM (2008) MetaMQAP: a meta-server for the quality assessment of protein models. BMC Bioinformatics 9: 403. 18823532 10.1186/1471-2105-9-403 PMC2573893

[47] Krieger E, Darden T, Nabuurs SB, Finkelstein A, Vriend G (2004) Making optimal use of empirical energy functions: force-field parameterization in crystal space. Proteins 57: 678–683. 15390263 10.1002/prot.20251

[48] Gu X (1999) Statistical methods for testing functional divergence after gene duplication. Mol Biol Evol 16: 1664–1674. 10605109 10.1093/oxfordjournals.molbev.a026080

[49] Gu X (2006) A simple statistical method for estimating type-II (cluster-specific) functional divergence of protein sequences. Mol Biol Evol 23: 1937–1945. 16864604 10.1093/molbev/msl056

[50] Doron-Faigenboim A, Stern A, Mayrose I, Bacharach E, Pupko T (2005) Selecton: a server for detecting evolutionary forces at a single amino-acid site. Bioinformatics 21: 2101–2103. 15647294 10.1093/bioinformatics/bti259

[51] Stern A, Doron-Faigenboim A, Erez E, Martz E, Bacharach E, et al (2007) Selecton 2007: advanced models for detecting positive and purifying selection using a Bayesian inference approach. Nucleic Acids Res 35: W506–511. 17586822 10.1093/nar/gkm382 PMC1933148

[52] Bakan A, Bahar I (2009) The intrinsic dynamics of enzymes plays a dominant role in determining the structural changes induced upon inhibitor binding. Proc Natl Acad Sci U S A 106: 14349–14354. 19706521 10.1073/pnas.0904214106 PMC2728110

[53] Bakan A, Meireles LM, Bahar I (2011) ProDy: protein dynamics inferred from theory and experiments. Bioinformatics 27: 1575–1577. 21471012 10.1093/bioinformatics/btr168 PMC3102222

[54] Atilgan AR, Durell SR, Jernigan RL, Demirel MC, Keskin O, et al (2001) Anisotropy of fluctuation dynamics of proteins with an elastic network model. Biophys J 80: 505–515. 11159421 10.1016/S0006-3495(01)76033-X PMC1301252

[55] Eyal E, Yang LW, Bahar I (2006) Anisotropic network model: systematic evaluation and a new web interface. Bioinformatics 22: 2619–2627. 16928735 10.1093/bioinformatics/btl448

[56] Sullivan JC, Kalaitzidis D, Gilmore TD, Finnerty JR (2007) Rel homology domain-containing transcription factors in the cnidarian Nematostella vectensis. Dev Genes Evol 217: 63–72. 17120026 10.1007/s00427-006-0111-6

[57] Gilmore TD, Wolenski FS (2012) NF-kappaB: where did it come from and why? Immunol Rev 246: 14–35. 22435545 10.1111/j.1600-065X.2012.01096.x

[58] Ernst MK, Dunn LL, Rice NR (1995) The PEST-like sequence of I kappa B alpha is responsible for inhibition of DNA binding but not for cytoplasmic retention of c-Rel or RelA homodimers. Mol Cell Biol 15: 872–882. 7823953 10.1128/mcb.15.2.872 PMC231969

[59] Pando MP, Verma IM (2000) Signal-dependent and -independent degradation of free and NF-kappa B-bound IkappaBalpha. J Biol Chem 275: 21278–21286. 10801847 10.1074/jbc.M002532200

[60] Huxford T, Huang DB, Malek S, Ghosh G (1998) The crystal structure of the IkappaBalpha/NF-kappaB complex reveals mechanisms of NF-kappaB inactivation. Cell 95: 759–770. 9865694 10.1016/s0092-8674(00)81699-2

[61] Jacobs MD, Harrison SC (1998) Structure of an IkappaBalpha/NF-kappaB complex. Cell 95: 749–758. 9865693 10.1016/s0092-8674(00)81698-0

[62] Malek S, Huang DB, Huxford T, Ghosh S, Ghosh G (2003) X-ray crystal structure of an IkappaBbeta x NF-kappaB p65 homodimer complex. J Biol Chem 278: 23094–23100. 12686541 10.1074/jbc.M301022200

[63] Taylor JS, Raes J (2004) Duplication and divergence: the evolution of new genes and old ideas. Annu Rev Genet 38: 615–643. 15568988 10.1146/annurev.genet.38.072902.092831

[64] Michel F, Soler-Lopez M, Petosa C, Cramer P, Siebenlist U, et al (2001) Crystal structure of the ankyrin repeat domain of Bcl-3: a unique member of the IkappaB protein family. EMBO J 20: 6180–6190. 11707390 10.1093/emboj/20.22.6180 PMC125740

[65] Jones DT (1999) Protein secondary structure prediction based on position-specific scoring matrices. J Mol Biol 292: 195–202. 10493868 10.1006/jmbi.1999.3091

[66] Tousignant A, Pelletier JN (2004) Protein motions promote catalysis. Chem Biol 11: 1037–1042. 15324804 10.1016/j.chembiol.2004.06.007

[67] Chu ZL, McKinsey TA, Liu L, Qi X, Ballard DW (1996) Basal phosphorylation of the PEST domain in the I(kappa)B(beta) regulates its functional interaction with the c-rel proto-oncogene product. Mol Cell Biol 16: 5974–5984. 8887627 10.1128/mcb.16.11.5974 PMC231600

[68] Whiteside ST, Epinat JC, Rice NR, Israel A (1997) I kappa B epsilon, a novel member of the I kappa B family, controls RelA and cRel NF-kappa B activity. EMBO J 16: 1413–1426. 9135156 10.1093/emboj/16.6.1413 PMC1169738

[69] Dehal P, Boore JL (2005) Two rounds of whole genome duplication in the ancestral vertebrate. PLoS Biol 3: e314. 16128622 10.1371/journal.pbio.0030314 PMC1197285

[70] Gu X, Wang Y, Gu J (2002) Age distribution of human gene families shows significant roles of both large- and small-scale duplications in vertebrate evolution. Nat Genet 31: 205–209. 12032571 10.1038/ng902

[71] Van de Peer Y (2004) Computational approaches to unveiling ancient genome duplications. Nat Rev Genet 5: 752–763. 15510166 10.1038/nrg1449

[72] Bork P (1993) Hundreds of ankyrin-like repeats in functionally diverse proteins: mobile modules that cross phyla horizontally? Proteins 17: 363–374. 8108379 10.1002/prot.340170405

[73] Lynch M, Conery JS (2000) The evolutionary fate and consequences of duplicate genes. Science 290: 1151–1155. 11073452 10.1126/science.290.5494.1151

[74] Lynch M, Force A (2000) The probability of duplicate gene preservation by subfunctionalization. Genetics 154: 459–473. 10629003 10.1093/genetics/154.1.459 PMC1460895

[75] Escriva H, Bertrand S, Germain P, Robinson-Rechavi M, Umbhauer M, et al (2006) Neofunctionalization in vertebrates: the example of retinoic acid receptors. PLoS Genet 2: e102. 16839186 10.1371/journal.pgen.0020102 PMC1500811

[76] Harrison RW (1984) Variational calculation of the normal modes of a large macromolecule: methods and some initial results. Biopolymers 23: 2943–2949. 6525408 10.1002/bip.360231216

[77] Yildirim Y, Doruker P (2004) Collective motions of RNA polymerases. Analysis of core enzyme, elongation complex and holoenzyme. J Biomol Struct Dyn 22: 267–280. 15473702 10.1080/07391102.2004.10507000

[78] Brooks B, Karplus M (1985) Normal modes for specific motions of macromolecules: application to the hinge-bending mode of lysozyme. Proc Natl Acad Sci U S A 82: 4995–4999. 3860838 10.1073/pnas.82.15.4995 PMC390485

[79] Marques O, Sanejouand YH (1995) Hinge-bending motion in citrate synthase arising from normal mode calculations. Proteins 23: 557–560. 8749851 10.1002/prot.340230410

[80] Tama F, Sanejouand YH (2001) Conformational change of proteins arising from normal mode calculations. Protein Eng 14: 1–6. 11287673 10.1093/protein/14.1.1

[81] Tama F, Brooks CL 3rd (2005) Diversity and identity of mechanical properties of icosahedral viral capsids studied with elastic network normal mode analysis. J Mol Biol 345: 299–314. 15571723 10.1016/j.jmb.2004.10.054

[82] Hayden MS, Ghosh S (2008) Shared principles in NF-kappaB signaling. Cell 132: 344–362. 18267068 10.1016/j.cell.2008.01.020

[83] Hoffmann A, Levchenko A, Scott ML, Baltimore D (2002) The IkappaB-NF-kappaB signaling module: temporal control and selective gene activation. Science 298: 1241–1245. 12424381 10.1126/science.1071914

