# Supplementary figures and images for: Evolutionary, Structural and Functional Interplay of the IκB Family Members
Source: PLoS One. 2013 Jan 23;8(1):e54178. doi: 10.1371/journal.pone.0054178 (PMC3553144; doi:10.1371/journal.pone.0054178)

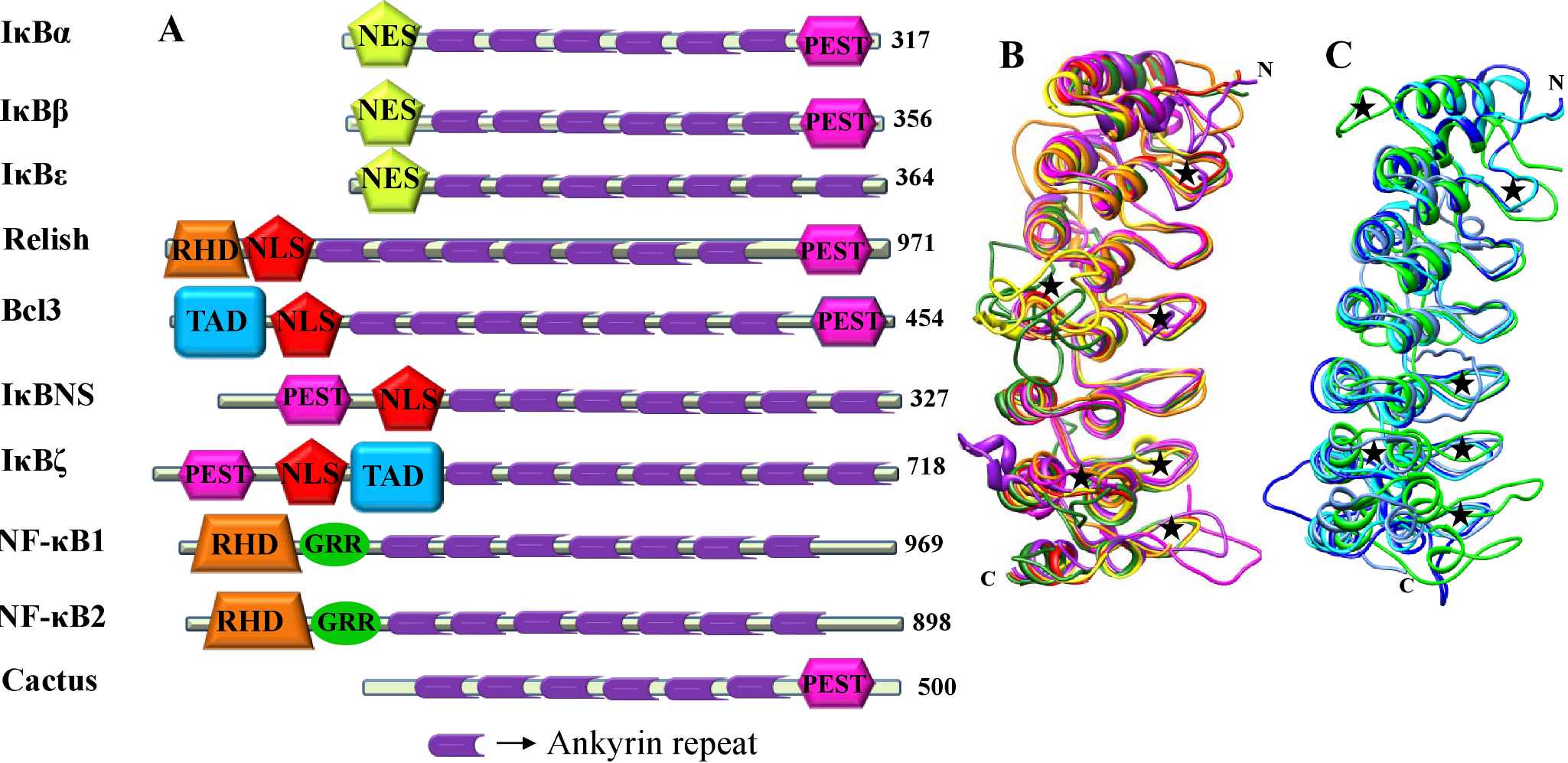

Supplement: Figure S1 — Domain organization and structural superimposition of IκB family members. (A) Domain organizations of the IκB subfamily members are shown. The number of amino acids in each protein is indicated on the right. NES, nuclear export sequence; NLS, nuclear localization signal; TAD, transactivation domain; DD, death domain; CC, coiled-coil domain; RHD, Rel-containing homology domain; and GRR, glycine-rich repeat. (B) Structural superimposition of typical IκB proteins and (C) IκB-like domain containing proteins. Major variations are shown by black stars in the ribbon representation of the IκB subfamily members. IκB proteins are colored as follows: IκBα – magenta; IκBβ – orange; IκBε – purple; IκBζ – forest green; IκBNS – yellow; Bcl3 – red; Cactus – cornflower blue; Relish – green; NF-κB1 – dark blue; and NF-κB2 – cyan. (TIF) [file pone.0054178.s001.tif]

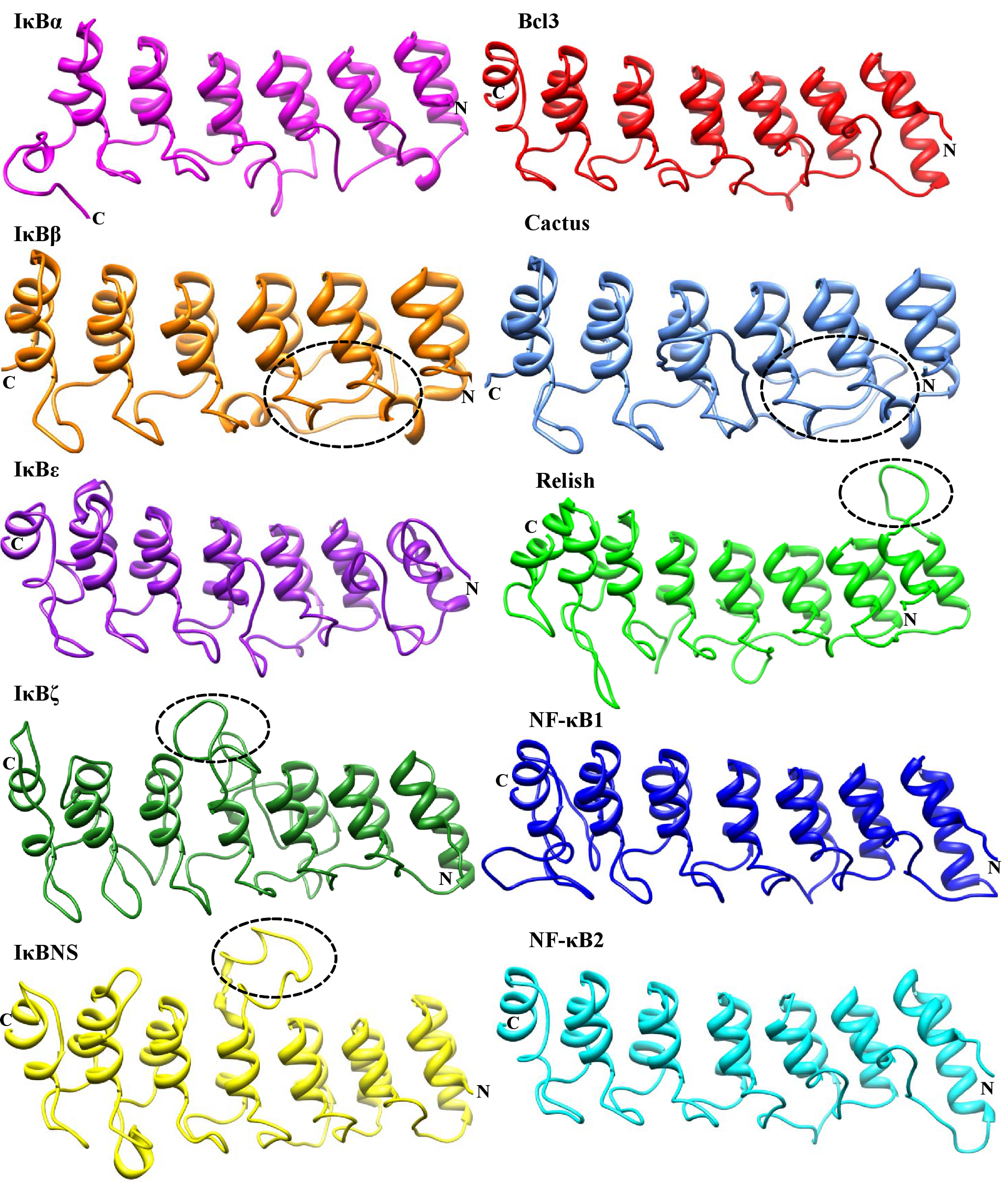

Supplement: Figure S2 — Comparative models of IκB proteins. Crystallographic structures of IκB proteins such as IκBα, IκBβ and Bcl3, are shown in ribbon representation. Other IκB proteins such as IκBε, IκBζ, IκBNS, Relish, Cactus, NF-κB1 and NF-κB2, are modeled structures. IκB proteins are colored as follows: IκBα – magenta; IκBβ – orange; IκBε – purple; IκBζ – forest green; IκBNS – yellow; Bcl3 – red; Cactus – cornflower blue; Relish – green; NF-κB1 – dark blue; and NF-κB2 – cyan. The insertion regions in the modeled and crystal structures are highlighted with dotted circles. (TIF) [file pone.0054178.s002.tif]

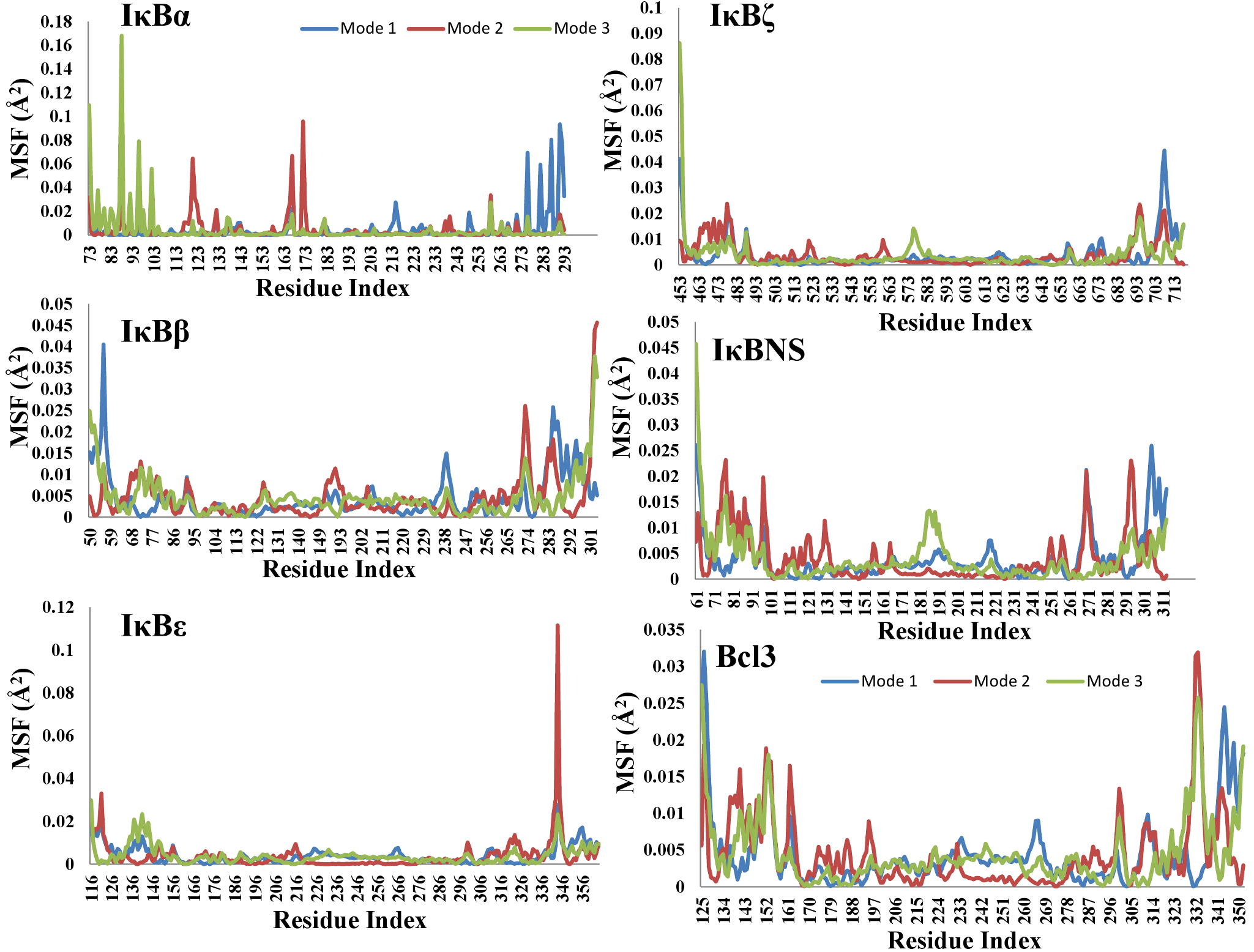

Supplement: Figure S3 — Fluctuation profiles of the 3 lowest frequency normal modes for the IκB subfamilies. The 3 lowest frequency normal modes obtained for the IκB proteins using ANM are shown. Fluctuation profiles have been shown as a function of residue number corresponding to the 3 lowest frequency modes. Mode 1 is shown in blue, mode 2 in red, and mode 3 in green. (TIF) [file pone.0054178.s003.tif]
