# Supplementary material for: Evolutionary, Structural and Functional Interplay of the IκB Family Members
Source: PLoS One. 2013 Jan 23;8(1):e54178. doi: 10.1371/journal.pone.0054178 (PMC3553144; doi:10.1371/journal.pone.0054178)
Supplement: Table S2 — PDB structures. The PDB structures that were utilized in our PCA analysis were resolved at 1.2 Å or higher. Bcl3 (PDB ID: 1K1A) was used as the reference structure. The numbers next to the PDB IDs indicate the number of individual NMR ensembles. (DOC) [file pone.0054178.s005.doc]

| **PDB IDENTIFIER** | **EXPERIMENTAL METHOD** | **RESOLUTION (Å)** |
| --- | --- | --- |
| 2BKG | X-RAY DIFFRACTION | 1.90 |
| 2BKK | X-RAY DIFFRACTION | 2.15 |
| 1OY3 | X-RAY DIFFRACTION | 2.05 |
| 2DVW | X-RAY DIFFRACTION | 2.30 |
| 2L6B(20) | SOLUTION NMR | - |
| 2F8X | X-RAY DIFFRACTION | 3.25 |
| 1NFI | X-RAY DIFFRACTION | 2.70 |
| 2XEH | X-RAY DIFFRACTION | 1.81 |
| 1TR4(20) | SOLUTION NMR | - |
| 2HE0 | X-RAY DIFFRACTION | 1.90 |
| 3AJI | X-RAY DIFFRACTION | 2.05 |
| 2XEE | X-RAY DIFFRACTION | 2.10 |
| 3B7B | X-RAY DIFFRACTION | 2.99 |
| 1YYH | X-RAY DIFFRACTION | 1.90 |
| 2V5Q | X-RAY DIFFRACTION | 2.30 |
| 1SVX | X-RAY DIFFRACTION | 2.24 |
| 3NBN | X-RAY DIFFRACTION | 3.45 |
| 1MJ0 | X-RAY DIFFRACTION | 2.03 |
| 2F8Y | X-RAY DIFFRACTION | 1.55 |
| 2QYJ | X-RAY DIFFRACTION | 2.05 |
| 2DWZ | X-RAY DIFFRACTION | 2.40 |
| 1N0Q | X-RAY DIFFRACTION | 1.26 |
| 1N0R | X-RAY DIFFRACTION | 1.50 |
| 1IKN | X-RAY DIFFRACTION | 2.30 |
| 1N11 | X-RAY DIFFRACTION | 2.70 |
| 3Q9N | X-RAY DIFFRACTION | 2.00 |
| 2P2C | X-RAY DIFFRACTION | 3.24 |
| 1K1B | X-RAY DIFFRACTION | 1.90 |
| 1K1A | X-RAY DIFFRACTION | 1.86 |
| 1K3Z | X-RAY DIFFRACTION | 2.50 |
| 3Q9U | X-RAY DIFFRACTION | 2.30 |
| 2J8S | X-RAY DIFFRACTION | 2.54 |
| 1QYM | X-RAY DIFFRACTION | 2.80 |
| 3B95 | X-RAY DIFFRACTION | 2.99 |
| 3NOC | X-RAY DIFFRACTION | 2.70 |
| 1UOH | X-RAY DIFFRACTION | 2.00 |
| 3NOG | X-RAY DIFFRACTION | 3.34 |
| 2QC9 | X-RAY DIFFRACTION | 2.35 |
| 2AJA | X-RAY DIFFRACTION | 2.80 |
| 3HRA | X-RAY DIFFRACTION | 1.69 |
| 2DZO | X-RAY DIFFRACTION | 3.00 |
| 2DZN | X-RAY DIFFRACTION | 2.20 |
| 2FO1 | X-RAY DIFFRACTION | 3.12 |
| 1WG0 | X-RAY DIFFRACTION | 2.53 |
| 1WDY | X-RAY DIFFRACTION | 1.80 |
| 1S70 | X-RAY DIFFRACTION | 2.71 |
| 1IXV | X-RAY DIFFRACTION | 2.30 |
| 1OT8 | X-RAY DIFFRACTION | 2.00 |
| 1MX2 | X-RAY DIFFRACTION | 2.25 |
| 1MX4 | X-RAY DIFFRACTION | 2.00 |
| 1MX6 | X-RAY DIFFRACTION | 2.00 |
| 1G3N | X-RAY DIFFRACTION | 2.90 |
| 1DC2(20) | SOLUTION NMR | - |
| 1DCQ | X-RAY DIFFRACTION | 2.10 |
| 1SW6 | X-RAY DIFFRACTION | 2.10 |
| 1BU9(21) | SOLUTION NMR | - |
| 1MYO(44) | SOLUTION NMR | - |
| 2MYO(1) | SOLUTION NMR | - |
| 1A5E(18) | SOLUTION NMR | - |
| 2A5E(1) | SOLUTION NMR | - |
| 1BLX | X-RAY DIFFRACTION | 1.90 |
| 1BI7 | X-RAY DIFFRACTION | 3.40 |
| 1BI8 | X-RAY DIFFRACTION | 2.80 |
| 1IHB | X-RAY DIFFRACTION | 1.95 |
| 1BD8 | X-RAY DIFFRACTION | 1.80 |
| 1AP7(20) | SOLUTION NMR | - |
| 1AWC | X-RAY DIFFRACTION | 2.15 |
| 1YCS | X-RAY DIFFRACTION | 2.20 |
| 3DEO | X-RAY DIFFRACTION | 1.50 |
| 3DEP | X-RAY DIFFRACTION | 2.70 |
| 3C5R | X-RAY DIFFRACTION | 2.00 |

*Residues 125 to 350 were included in the analysis, corresponding to 20% identity of Bcl-3 sequence.
